# Menstrual health communication among Indian adolescents: A mixed-methods study

**DOI:** 10.1371/journal.pone.0223923

**Published:** 2019-10-17

**Authors:** Mukta Gundi, Malavika A. Subramanyam

**Affiliations:** Indian Institute of Technology Gandhinagar, Gujarat, India; Università degli Studi di Perugia, ITALY

## Abstract

**Background:**

Research in health communication frequently views it as an information dissemination strategy, thus neglecting the intricacies involved in communicating a sensitive topic such as menstruation. The social patterning in menstrual communication, a taboo in India, and its consequent health-effects on adolescents are under-studied.

**Methods:**

We studied the social determinants of menstrual communication influencing menstrual- health through semi-structured interviews of 21 boys and girls each, 12 key-respondent interviews, followed by a cross-sectional survey of 1421 adolescents from Nashik district, India. We thematically analysed the qualitative data and fit multivariable logistic regression to model risk ratios.

**Findings:**

We found social disparities in adolescents’ experiences of communication taboo regarding menstruation. While boys curbed their curiosity about the topic, girls too faced resistance to their experience-sharing and treatment-seeking for menstrual illnesses. The inequality in menstruation-related communication was evident as more boys than girls faced avoidance to their questions [IRR at 95%CI: 2.75 (2.04, 3.71)]], and fewer tribal than rural girls were communicated severe taboos (OR at 95% CI: 0.18 (0.09, 0.36))]. Girls who had been communicated severe (versus no/mild) taboos reported greater stress about menstrual staining (IRR at 95% CI: 1.31 (1.10, 1.57)], emphasizing the health consequences of such communication inequalities.

**Conclusions:**

Our study highlights the need to address gender and setting-specific communication experiences of adolescents in India, a patriarchal society. The inequality in communication needs attention as it creates unequal patterns in Indian adolescents’ menstrual health and experiences, which may manifest as inequities in reproductive health-related outcomes even in their adult-lives.

## Introduction

### Background and literature review

Adolescents make up 20% of India’s population and face several sexual and reproductive health (SRH) challenges in this transformative age [1]. About 27% adolescent girls (“girls”) are married; one in six bear children; 10% adolescent boys (“boys”) experience premarital sex; yet many adolescents lack awareness regarding healthy behaviors and have unmet treatment-needs [1,2]. In addition to a rural-urban disparity in SRH, tight gender norms compound the effects of SRH outcomes, as only 40% of married girls have health-related autonomy, while 72% young men reported that women need their husband’s permission for most things in life [1]. Several *social* determinants such as age, gender; exposure to sex education; exposure to media; school-type; parental supervision, and parents’ educational level can influence SRH behaviors and outcomes [2–4]. One channel of their influence on SRH is through effective communication.

However, SRH communication remains a taboo, making it harder for adolescents to discuss sensitive topics, including menstruation [1,5–9]. Menstruation-related communication, largely considered a ‘women’s topic’, often remains a one-way prescriptive process for girls to receive abrupt and indirect messages from a same-sex parent or a teacher with a limited role of men [5,7,10–14]. Although Indian patriarchal mindset and sociocultural practices have been argued to inhibit menstruation-related communication; no Indian studies have focused on how various social determinants create patterns in interpersonal communication (“IP communication”) on menstruation and in turn, affect Indian adolescents’ health [8].

Although we found no studies on features of menstruation-related IP communication and its impact on Indian adolescents’ health, the extent to which adolescent health and lives get affected due to a lack of information is well established. For instance, girls avoid seeking treatment as they feel shy [15]; Karnataka boys reported viewing pornography to seek answers for SRH-related queries [4]; a qualitative study of Indian boys from three states showed that boys possessed a negative attitude towards menstruation [16]; and only 17.5% Patna boys (n = 461) were aware of the menstruation physiology as compared to 33.1% girls (n = 587) indicating significant gender difference (p<0.001) [2]. Thus, SRH-related communication either remains non-inclusive in terms of gender and socioeconomic setting or falls short of capturing the nuances of communication (and silence) [10,17]. Additionally, the contribution of various social determinants, defined by the World Health Organization as “the conditions in which people are born, grow, live, work and age”, in creating ‘health communication inequality’ is unexplored in the Indian context [18,19]. Therefore, this mixed-methods study attempts to explore the social determinants of menstruation-related IP communication among girls and boys from different socioeconomic settings, and their association with menstruation-related health outcomes. Among adolescents, the social factors or contexts such as neighborhoods, school, parental social and economic factors, social norms, gender, and by the distribution of money, power and resources at various levels are considered to be the determining factors of their health [20–22].

### Theoretical approach

**Fig 1:**
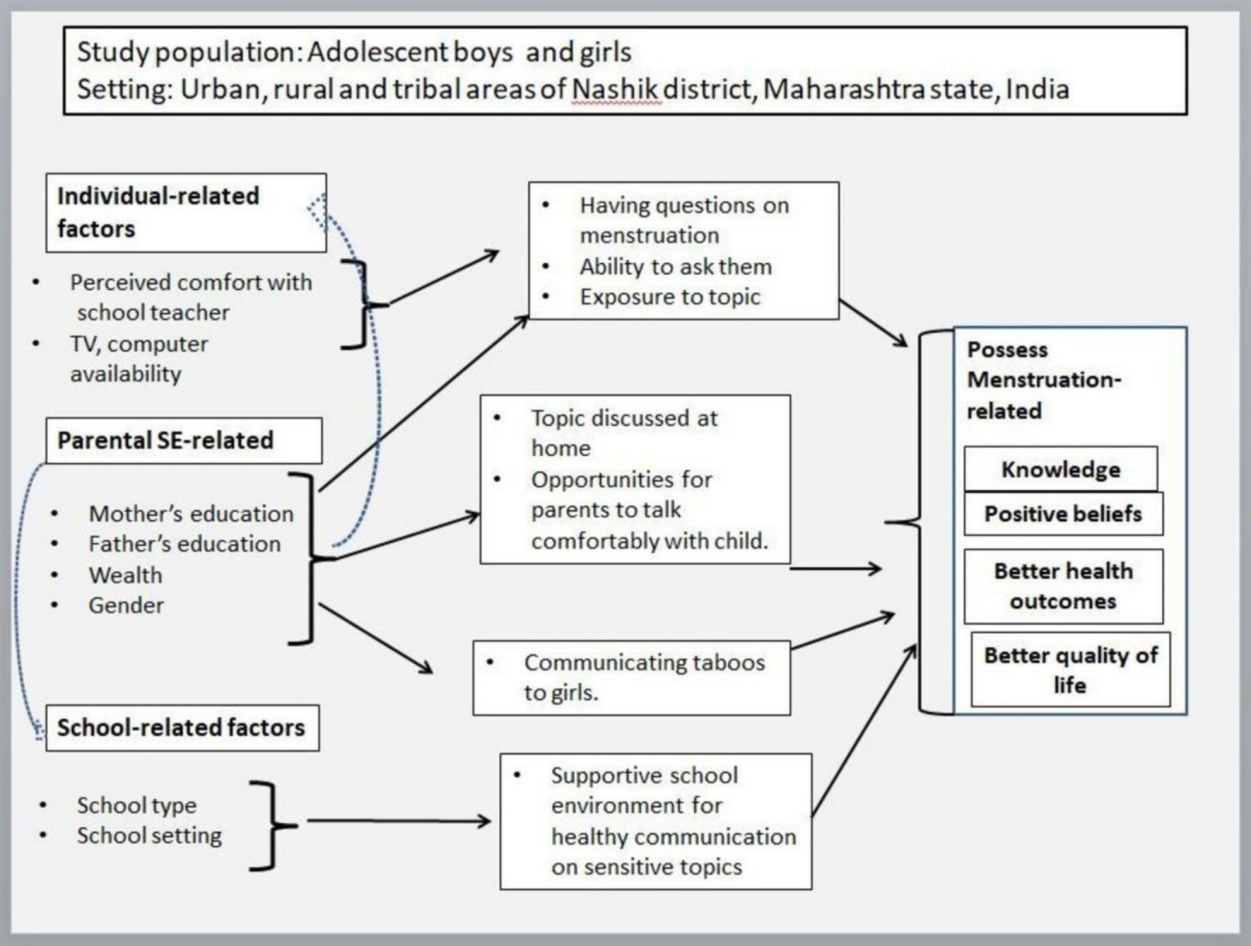
Conceptual model of the study.

We draw from the concept of communication inequality [23,24]. Communication inequality is *“differences in the generation*, *manipulation*, *and distribution of information among social groups; and differences in (a) access and use*, *(b) attention*, *(c) retention*, *and (d) capacity to act on relevant information among individuals*” [18]. However, in this study, we look at communication as a process beyond its use in the generation of and access to health information and additionally focus on how communication plays role in social construction of menstruation and associated taboos. Additionally, we study the association of social determinants in creating unequal communication patterns and their ability to influence health (Fig 1).

## Methods

As this is a sensitive topic to discuss with adolescents, we have taken utmost care in terms of ethics and confidentiality by adhering to the protocol approved by the IIT Gandhinagar Institutional Ethics Committee. Verbal consent was taken from responsible adults as well as from school authorities and the survey was anonymous. We refrained from taking a written consent from the responsible adults and participants as paperwork and signatures are frequently viewed with suspicion in the Indian context, and can damage the already established rapport. As we aimed at collecting the data on a sensitive topic, we wanted to gain their trust by ensuring utmost anonymity. In order to keep the process an informed one, we provided a written information sheet that explained the objective and the details of the study procedure to the responsible adults. When requested, we also shared our questionnaire draft with the school authorities. Once we received an assent from the school authorities, parents’ or guardians’ assent was received through parent-teacher meetings/ group text-messages/ face-to-face meetings. Schools permitted us to collect survey data only upon receiving parental assent. Prior to collecting the data, we sought verbal consent from each participant after explaining the study’s goals and procedure, and assuring them of confidentiality and anonymity. The Institutional Ethics Committee recommended that we seek assent from teachers and parents as well as verbal consent from the participants. We have taken care to adhere to the ethics process approved by the ethics committee.

### Study site

This study was carried out in Nashik district from Maharashtra state in India, which has 4th highest tribal population in the state [25,26]. It is known for its high Human Development Index (0.746), while conversely having inlands which are some of the poorest in the state [27].

### Study design

A sequential mixed methods design (Qual→ Quant→ qual) was followed for collecting the data from adolescents aged 13 to 19 years [28]. Semi-structured interviews and focus group discussions (“FGDs”) of adolescents and, key-respondent respondent interviews were followed by a school-based survey. A short qualitative phase was carried out at the end to seek respondents’ reflections on study findings.

#### Qualitative phase 1

Given the sensitive nature of the topic, we restricted our study to 13 to 19 year-old adolescents. We sampled the participants through a mix of snowball and purposive sampling to identify adolescents from three sub-district administrative divisions. Semi-structured interviews of 21 boys, 21 girls and 12 key-respondents (4 parents/ guardians, 3 teachers, 5 healthcare providers including a gynaecologist, an Ayurveda doctor, a homeopath, a nurse and a psychologist), along with nine FGDs with adolescents were carried out in a familiar yet private settings at schools, homes, or at the workplaces (with the exception of one FGD which was conducted in the premise of a local organization working on sanitation). Interviews taken using a semi-structured interview guide (Appendix A) lasting for approximately 25 to 45 minutes and FGDs lasting for 50–80 minutes were audio-taped. We did not tape three boys’ interviews and one girls’ FGD, as participants seemed nervous, and instead continued the discussion while taking active notes. During conversations, participants were slowly steered towards the topic after providing a general introduction. Data were collected until saturation was reached. Most of the interviews and FGDs of boys were carried out by a male interviewer, who has an extensive experience with health-related fieldwork in Maharashtra. For three interviews and three FGDs of boys, a female interviewer was present in addition to a male moderator. Perceived differences in their responses with and without the female interviewer were noted. Non-verbal responses and interviewers’ observations were noted, which helped in contextualizing our study. More details of the qualitative phase methodology are presented in Appendix B.

A survey question-bank based on the qualitative data was formed. Quantitative measures were developed by us based on our conceptual understanding of adolescents’ menstruation-related experiences guided by our field visits, observations, qualitative interviews and focus group discussions with adolescents and key informants, such as sex-education expert. On the basis of such formative research, we decided to collect the information about basic awareness, and communication-related experiences (such as: whether menstruation occurs among girls or boys; and, do you feel comfortable to ask menstruation-related question to your school-teacher). We checked the content validity of our survey questionnaire with content experts (public health expert, adolescent sex-education expert and a school teacher) prior to the survey to know if our survey captured foundational understanding and varied shades of menstruation-related experiences among adolescent girls and boys. As this was a self-administered school-based survey, we designed a questionnaire that was easy to understand and used simple and locally relevant language that had a sixth-grade reading level. The school teachers who reviewed the questionnaire confirmed this. When we field-tested the questionnaire on 35 boys and girls; each, including those from both low and high resource settings, we requested them to indicate any questions that were difficult to comprehend. Based on this feedback from our pre-test, a few unclear response options, complex sentences, were modified into simpler, multiple choice questions. The questionnaire was translated into Marathi; then back-translated into English, and its content validity was checked by language and translation experts. During data collection, we clarified participants’ questions or doubts regarding the survey questions. Fewer than ten participants in the entire sample expressed an inability to comprehend the questionnaire and we assisted them by reading the questions aloud.

#### Survey sampling and population

A stratified list of schools based on setting (urban/rural/tribal) and type (government/ private aided/ private unaided) was prepared for selecting adolescents from various socioeconomic settings [29]. The sample size for the survey was decided by calculating the maximum and the minimum expected prevalence for all possible study outcomes and by comparing it among all possible subgroups of adolescents using two-sided test with 80% power. The highest required sample size with 10% non-response was 1500, of which we could sample 1491 adolescents. The analytical sample of 1421 was arrived at after excluding data of 70 premenarchal girls.

After school authorities assented, schools representatives were requested to acquire verbal consent from students’ parents/ guardians. Survey data was collected from 27 out of 35 schools which were initially approached (based on snowball sampling), covering about 2.5% of secondary schools in Nashik [30]. To minimize bias, students with alternate roll numbers or from alternate seats were chosen from different educational divisions. Smooth administration of the survey was ensured by a research assistant (“RA”) and a school staff-member who closely monitored the survey. The RA read out the questions and survey options aloud for students with reading difficulty.

In our survey, we captured data on various social determinants that can potentially impact adolescents’ menstrual health communication. These include: School-related determinants: school setting and funding type; socioeconomic background related determinants: parental education, household wealth and occupation, individual-related determinants: gender and age of the participant. Socioeconomic predictors were: school setting (urban/rural/tribal) and funding type (government/private aided/private unaided), parental education (no or some elementary schooling/ middle-school/ high-school or some college/ completed college or more), household wealth (poorest/middle/richest tertile) and gender (girls/boys). Communication-related variables were: asking question regarding menstruation to family (yes/no), comfort to ask menstruation-related question to teacher (complete comfort/lack), facing avoidance to menstruation-related question from family (yes/no) and taboos being communicate (no or mild/moderate/severe). Health outcomes were: knowledge (possess/not), beliefs (positive/not) regarding menstruation among adolescents, menstrual health status (poor/moderate/good), stress regarding stain (yes/no), missed school during periods (yes/no) among girls. The details of predictors, covariates and outcomes are given in Appendix C.

#### Post-survey qualitative phase

Semi-structured interviews of 5 girls (of which, 2 were married), one FGD with school-dropout girls and three key respondents (a female tribal-village head, a sex education expert and a social worker) were carried out in the post-survey qualitative phase, in which we sought their opinions on our primary findings. We could not include school dropout boys due to lack of consent from their parents.

#### Ethical considerations

Ethical approval was obtained from the IIT Gandhinagar Institutional Ethics Committee before commencing the study. The survey objectives and procedure was discussed with school authorities. One urban private unaided school asked us to first seek permission from the psychologist appointed by the school, before beginning the study. Several school administrators across all settings requested us to share a sample questionnaire, upon reviewing which we were given/denied permission to conduct the survey. We received rejection from eight out of 35 approached schools across different settings. Although most schools did not share with us the specific reasons for rejection, a couple of schools indicated that inclusion of boys in a study on menstruation was a reason. As per the suggestions given by the school authorities, prior verbal consent was obtained from parents/guardians by the school-staff. After obtaining consent, school authorities invited us to administer the survey. We shared the study purpose, the voluntary, and the anonymous nature of participation, and requested participants’ verbal consent by assuring confidentiality. Students were informed by the teachers and by the data collectors that the survey was not a graded academic assignment. They were assured that even if they wanted to quit the study at any given point, their academic performance would not be affected in any way. Students had the option of selecting either an English or Marathi survey form. Parental assent from the participants of the qualitative study was directly taken by MG in their homes or by teachers who introduced the participants to MG. This was followed by seeking verbal consent from the adolescent participants. During one of the focus group discussions in an urban setting, a 14 year old adolescent boy did not participate after he expressed a wish to opt out of the discussion because he felt he was discussing a topic that was “inappropriate to discuss.” He was gently led to a separate space and within minutes was watched by an adolescent psychologist to ensure his emotional wellbeing. The psychologist was contacted by the researcher later to confirm the wellbeing of the said participant. There were no other instances of participants reporting any form of distress.

### Analysis

#### Qualitative data analysis

The data recordings were transcribed and translated into English. An expert verified the translations to ensure that nuanced meanings were preserved. Qualitative data was analyzed using a thematic analysis [31]. We identified codes from the written transcripts of the semi-structured interviews and focus group discussions using an iterative process—wherein girls’ and boys’ day-to-day experiences, observations regarding menstruation; their own understanding of communication and silence regarding this topic, and their narratives about communication taboo and menstrual health helped and guided the multiple stages of coding, and analysis. The coders also added their own individual reflections, and codes, guided by the thick notes collected during data collection, as they tried to gain a deeper understanding of the nuances of interpersonal communication or a lack of that regarding menstruation. By identifying semantic codes (that summarize the content of qualitative data) and later, the interpretative codes (that describe the underlying interpreted story of the data), we arrived at the list of themes and subthemes. As a process of triangulation, codes and themes were compared among coders. Upon achieving full agreement on final codes and findings through a thorough discussion we decided to use these results to guide the drafting of our survey question bank. Member (participant) rechecking, peer checking, deep engagement with the participants and key respondent interviewing also strengthened the triangulation process [32].

#### Quantitative data analysis

Descriptive statistics of the main variables (Tables 1 and 2) were computed after data cleaning and labelling. So as to capture adolescents’ knowledge, beliefs and taboo-related communication experiences, we created composite measures (See Appendix C) by using very few variables in each index (where items are cause indicators) [33]. We used Poisson regression models with survey commands to model incidence rate ratios (equivalent to prevalence ratios in our study) of the binary dependent variables. Alpha was set at 0.05. Robust standard errors were used in the regression analysis to account for school-level clustering. Generalized ordered logit models with survey commands were fit with the ordinal dependent variable to yield odds ratios. An unadjusted model (Model 1), with later adjustment for age, setting and gender (Model 2) followed by additional adjustment for school type and/or variables indicating parental socioeconomic status (Model 3) were fit. We tested the sensitivity of our findings to the exclusion of non-significant predictors. Appendix D gives a detailed list of the questions from the questionnaire that was used for the analysis. Data analyses were performed using STATA version 12 [34].

**Table 1:** Distribution of communication-related outcomes among adolescents across social determinants (Row percentages) (n = 744 boys and 677 girls).

** **	**Girls**								**Boys**							
** **	Comfort to ask questions to school teacher				Ever asked a question to family		Faced avoidance by family		Comfort to ask questions to school teacher				Ever asked a question to family		Faced avoidance by family	
	Not at all	Gender dependent	Completely	**Total**	Yes	**Total**	Yes	**Total**	Not at all	Gender dependent	Completely	**Total**	Yes	**Total**	Yes	**Total**
Setting																
**Rural**	50	64	4	118	70	119	12	70	0	84	33	117	18	118	14	18
**%**	**42.37**	**54.24**	**3.39**	**100**	58.82	**100**	**17.14**	**100**	**0**	**71.79**	**28.21**	**100**	**15.25**	**100**	**77.78**	**100**
**Tribal**	35	151	27	213	**157**	211	**55**	153	1	125	73	199	60	196	46	60
**%**	**16.43**	**70.89**	**12.68**	**100**	74.41	**100**	35.95	**100**	**0.50**	**62.81**	**36.68**	**100**	**30.61**	**100**	**76.67**	**100**
**Urban**	82	189	58	329	195	339	**56**	**195**	91	199	112	402	39	406	**23**	**39**
**%**	**24.92**	**57.45**	**17.63**	**100**	57.52	**100**	28.72	**100**	**22.64**	**49.50**	**27.86**	**100**	**9.61**	**100**	**58.97**	**100**
School Type																
**Government**	35	143	33	211	150	210	55	150	0	132	63	195	61	197	44	61
**%**	**16.59**	**67.77**	**15.64**	**100**	71.43	**100**	**36.67**	**100**	**0**	**67.69**	**32.31**	**100**	**30.96**	**100**	**72.13**	**100**
**Private Aided**	107.00	165	33	348	211	355	42	207	53	233	95	381	39	381	31	39
**%**	**30.75**	**47.41**	**9.48**	**100**	59.44	**100**	**20.29**	**100**	**13.91**	**61.15**	**24.93**	**100**	**10.24**	**100**	**79.49**	**100**
**Private Unaided**	25	53	23	101	61	104	26	61	39	42	60	141	17	141	8	17
**%**	**24.75**	**52.48**	**22.77**	**100**	58.65	**100**	**42.62**	**100**	**27.66**	**29.79**	**42.55**	**100**	**12.06**	**100**	**47.06**	**100**
Wealth Tertile																
**Poorest**	43	130	23	196	138	200	41	136	6	138	73	217	54	213	38	54
**%**	**21.94**	**66.33**	**11.73**	**100**	69.00	**100**	**30.15**	**100**	**2.76**	**63.59**	**33.64**	**100**	**25.35**	**100**	**70.37**	**100**
**Middle**	60	116	21	197	120	201	28	120	32	137	50	219	22	224	20	22
**%**	**30.46**	**58.88**	**10.66**	**100**	59.70	**100**	**23.33**	**100**	**14.61**	**62.56**	**22.83**	**100**	**9.82**	**100**	**90.91**	**100**
**Richest**	44	116	36	196	120	201	32	120	49	88	76	213	25	212	12	25
**%**	**22.45**	**59.18**	**18.37**	**100**	59.70	**100**	**26.67**	**100**	**23.00**	**41.31**	**35.68**	**100**	**11.79**	**100**	**48.00**	**100**
Mothers Education																
**No/Some elementary schooling**	59	173	27	259	171	257	55	168	6	170	86	262	55	259	44	55
**%**	**22.78**	**66.80**	**10.42**	**100**	66.54	**100**	**32.74**	**100**	**2.29**	**64.89**	**32.82**	**100**	**21.24**	**100**	**80.00**	**100**
**Some middle school**	64	100	28	192	128	199	31	127	41	144	52	237	36	237	25	36
**%**	**33.33**	**52.08**	**14.58**	**100**	64.32	**100**	**24.41**	**100**	**17.30**	**60.76**	**21.94**	**100**	**15.19**	**100**	**69.44**	**100**
**Completed high-school/Some college**	30	60	10	100	63	104	19	63	27	59	32	118	9	122	4	9
**%**	**30.00**	**60.00**	**10.00**	**100**	60.58	**100**	**30.16**	**100**	**22.88**	**50.00**	**27.12**	**100**	**7.38**	**100**	**44.44**	**100**
**Completed college or more**	11	65	20	96	51	96	14	51	16	29	40	85	13	85	7	13
**%**	**11.46**	**67.71**	**20.83**	**100**	53.13	**100**	**27.45**	**100**	**18.82**	**34.12**	**47.06**	**100**	**15.29**	**100**	**53.85**	**100**
Fathers Education																
**No/Some elementary schooling**	38	116	19	173	122	175	37	121	1	104	56	161	37	160	31	37
**%**	**21.97**	**67.05**	**10.98**	**100**	69.71	**100**	**30.58**	**100**	**0.62**	**64.60**	**34.78**	**100**	**23.13**	**100**	**83.78**	**100**
**Some middle school**	57	120	22	199	132	200	40	129	31	157	55	243	36	243	26	36
**%**	**28.64**	**60.30**	**11.06**	**100**	66.00	**100**	**31.01**	**100**	**12.76**	**64.61**	**22.63**	**100**	**14.81**	**100**	**72.22**	**100**
**Completed high-school/Some college**	40	71	16	127	73	132	21	72	26	88	39	153	17	154	13	17
**%**	**31.50**	**55.91**	**12.60**	**100**	55.30	**100**	**29.17**	**100**	**16.99**	**57.52**	**25.49**	**100**	**11.04**	**100**	**76.47**	**100**
**Completed college or more**	27	86	26	139	80	140	17	80	34	51	60	145	24	147	12	24
**%**	**19.42**	**61.87**	**18.71**	**100**	57.14	**100**	**21.25**	**100**	**23.45**	**35.17**	**41.38**	**100**	**16.33**	**100**	**50.00**	**100**
Gender																
**Girls**	167	404	89	660	422	669	123	418								
**%**	**25.30**	**61.21**	**13.48**	**100**	**63.08**	**100**	**29.43**	**100**								
**Boys**									92	408	218	718	603	720	83	117
**%**									**12.81**	**56.82**	**30.36**	**100**	**83.75**	**100**	**70.94**	**100**

Missing values (%): Knowledge (2.97), Ever asked a question (2.25), Faced avoidance (7.29), Comfort with teacher (3.12), School Type (0.07), Setting (0), Mother's Education (2.30), Father's Education (3.12), Wealth Tertile (12.07), Beliefs (12.96), Menstrual Health Status (6.79), Stress regarding stain (1.32), School missed (1.32), Taboos communicated (4.87)

**Table 2:** Distribution of health-related outcomes among adolescents across communication factors (Row percentages) (n = 744 boys and 677 girls).

** **	**Boys**		**Girls**			
** **	Knowledge regarding menstruation					
Ever asked a question to family	**Possess knowledge**	Total	**Possess knowledge**	Total		
**No**	69	578	74	247		
**%**	**11.94**	**100**	**29.96**	**100**	** **	** **
**Yes**	18	105	158	422		
**%**	**17.14**	**100**	**37.44**	**100**	** **	** **
Comfort to ask question to school teacher						
**Not at all comfortable**	8	88	43	167		
**%**	**9.09**	**100**	**25.75**	**100**	** **	** **
**Comfort depends on gender of the teacher**	32	388	152	404		
**%**	**8.25**	**100**	**37.62**	**100**	** **	** **
**Completely comfortable**	47	205	36	89		
**%**	**22.93**	**100**	**40.45**	**100**	** **	** **
** **	**Beliefs regarding menstruation**					
** **	**Positive beliefs**	**Total**	**Positive beliefs**	**Total**	** **	
Ever asked a question to family						
**No**	116	571	23	210		
**%**	**20.32**	**100**	**10.95**	**100**		
**Yes**	36	100	56	359		
**%**	**36.00**	**100**	**15.60**	**100**		
Faced avoidance to question asked						
**No**	15	29	47	252		
**%**	**51.72**	**100**	**18.65**	**100**		
**Yes**	21	71	9	103		
**%**	**36.00**	**100**	**8.74**	**100**		
** **			**Stress regarding menstrual Stain**			
Taboos being communicated			**Have stress**	**Total**		
**No**			12	27		
%			**44.44**	**100**		
**Mild**			130	316		
%			**41.14**	**100**		
**Moderate**			46	145		
%			**31.72**	**100**		
**Severe**			45	149		
**%**			**30.20**	**100**		
** **			**School missed during periods**			
Taboos being communicated			**Yes**	**Total**		
**No**			4	27		
%			**14.81**	**100**		
**Mild**			72	317		
%			**22.71**	**100**		
**Moderate**			41	147		
%			**27.89**	**100**		
**Severe**			36	148		
%			**24.32**	**100**		
** **			**Menstrual health status**			
Taboos being communicated			**Poor**	**Moderate**	**Good**	**Total**
**No**			9	11	6	26
%			**34.62**	**42.31**	**23.08**	**100**
**Mild**			154	118	34	306
%			**50.33**	**38.56**	**11.11**	**100**
**Moderate**			67	62	9	138
%			**48.55**	**44.93**	**6.52**	**100**
**Severe**			67	57	19	143
%			**46.85**	**39.86**	**13.29**	**100**

## Results

### Qualitative findings

The themes emerging from our pre-survey qualitative phase are based on several instances of adolescents experiencing resistance against menstruation-related communication. Many were forced to use non-verbal modes of discussing the topic and experienced silence, which appeared to affect their menstruation-related experiences and health. Our themes described below also showcase how girls’ and boys’ communication-related experiences tended to differ, further deepening the differences in their menstruation-related understanding and beliefs. Overall, boys as compared to girls, and disadvantaged adolescents as compared to their wealthier counterparts, seemed to find menstruation-related IP communication more difficult. Our qualitative data also described how these communication barriers influenced girls’ health-related experiences and healthcare seeking. Boys’ communication barriers also seemed to reduce their opportunities to discuss menstruation openly, further affecting their understanding and beliefs. We protect participant confidentiality by including anonymous quotes.

#### Resistance to communication

Although both girls and boys found it difficult to discuss menstruation, few boys reported being scolded for talking about it as reflected in 15-years old rural boy’s interview:

“*I knew that my sister and mother follow some rituals for few days each month*. *I used to ask them about it but they would avoid the topic*. . . .*So*, *one day I brought a book home and was going through the pictorial illustrations on menstruation to understand what it is*. *My sister confronted me*. *I told her that I was reading about menstruation… she scolded me…*”

Any attempt to question taboos was reported to be ignored. Several girls were told ‘*how to behave*’, and ‘*how to use an absorbent’* upon reaching menarche. No girls, with an exception of a few from wealthy educated urban families, reported a healthy discussion on the topic or participation of men. Couple of girls from disadvantaged backgrounds were told to accept menstrual illness as “*a natural consequence of periods”*, belittling their effort to communicate their symptoms. Adults’ resistance reflected in this quote from an urban school-teacher:

“*We always invite parents when we teach ‘the story of my life’ to their teenage kids*. *Only a few parents*, *who are well aware of such issues*, *turn up*. *Some parents even resist the idea of such workshops*.*”*

The resistance to communicate combined with taboos and parents’ lack of awareness regarding healthy menstruation seemed to take a toll on girls’ reproductive health. Although hiding menstrual symptoms was not common among urban girls from educated families, several urban girls from lower socioeconomic background refused to reveal their menstrual complaints (including itching, dryness, excess white discharge) to anyone, especially to a male doctor. This describes how communication taboo can potentially influence their health. Girls from urban low resource setting preferred to keep those complaints hidden or preferred home-based treatment as they felt shame in describing their complaints. Resistance in communicating their menstrual complaints seemed to create fear, stress and body-shame among girls, as revealed in the following quote from a 13-year old urban girl living in an urban slum-

“*Several times I get white discharge*.. *However*, *I don’t tell this to my mom*.. *I don’t even tell her when I get my periods*.. *Better to keep it a secret*.. *I don’t like periods… My friend has suggested that if I don’t want periods ever in my life then I should take some home-made ayurvedic kadha (medication) that can stop it permanently*. *Can I do that*? *Can you give me more information about it*? *I don’t want periods…”*

Although such inequality in the communication-experience was evident, one of our key respondents, an urban school teacher and an experienced sex-education expert who had delivered several workshops in various socioeconomic settings, shared that innovative methods such as monoacts, *prasang-natya* (interactive theatrical exercise) and creative sessions on emotional intelligence and gender sensitization could help overcome the barriers in menstrual health communication even among disadvantaged adolescents. An educated parent from an urban area emphasized that parents’ group sessions with their children designed for discussions on menstruation would facilitate communication within families and in the community. However, in rural and tribal areas, menstruation-related education was believed to be the responsibility of school teachers. A school-appointed adult guardian from a tribal setting pointed out that the resistance to talking about sensitive topics within families is also coupled with the inability of parents to prioritize this discussion as they struggle with poverty, indicating how communication-related inequalities could stem from social inequalities. She added,

“*We have to fight with parents so that their daughters remain enrolled in school and not get married*. *Several tribal parents are reluctant to provide minimal fees that residential schools charge for their daughters*. *Poverty and constraints related to it make these girls’ lives complex*. *How can we expect parents to discuss something so sensitive with their children*?”

Our interviews with healthcare professionals revealed that their approach towards menstruation remained restricted to menstruation-related pathologies and clinically availed treatments.

#### Nonverbal communication, silence and euphemism

Many boys curbed their curiosity due to an unsaid understanding in the family about ‘*what not to talk*’ and girls narrated instances when they used euphemism to communicate. Using phrases such as *“aunty has arrived”*, *“she is sitting away”* or *“a crow has touched her”* was a common practice in all settings. Several girls narrated their experience of communication-taboo: several were instructed to use a sanitary absorbent with a “*keep it down there*” and an accompanied eye-signal; while a rural girl received a prescription for menstrual illness from a “lady-doctor” in silence, with no mention of menstruation. A majority of boys experienced menstrual taboos at home; however, a few experienced them outside. Following experience of a 16-years old urban boy captures how they realize the taboos:

“*We had a school initiative through which each of us worked in various shops in the city*. *I was working at a pharmacy*. *A lady came in and asked for sanitary pads*. *I showed her 2–3 packs of the brand and asked her casually ‘which one would you like to buy*?*’ She was a little awkward*. *The pharmacist saw that*, *came in hurriedly*, *pushed me aside*, *silently packed one of the packs and sent me inside* …*”*

Poor urban girls experienced untouchability and shame from the neighbours as they had to share a public toilet. They suffered from menstrual illnesses due to unclean toilet. Pre-menarcheal silence from adults left many urban poor and rural girls feeling unprepared and anxious at menarche. Interviewers often experienced silence as adolescents, especially rural and tribal boys took long pauses to answer and open-up in interviews. Girls from wealthy urban families and girls who had discussed menstruation with their mothers were observed to be more comfortable and confident in the interviews and focus group discussions.

School teacher’s body language and gestures were observed to play an important role in sustaining the silence around menstruation. Female teachers from urban schools pointed out that although both men and women feel awkward to talk about this topic, male teachers’ negative attitude towards this topic becomes obvious. A female teacher from an urban school which is attended by mostly students from low resource settings narrated an incident-

“*We (female teachers) were taking a health education class for adolescent students in our school*. *As soon as we started talking about menstruation*, *all male teachers left the room silently and locked the classroom door from outside*. *Wouldn’t students have seen this*?*”*

From our interviews with adolescents as well as key respondents we learned that an ability to explicitly use and listen to words describing female body parts such as vagina, and body processes such as menstruation, was missing among several participants as well as their school teachers and parents. A highly educated mother of an urban boy asked, “*Nobody told me about this when I was his age*, *did I lose anything*?” Such justification seemed to contribute towards sustaining the silence around this topic, especially for boys.

### Quantitative survey findings

Our data brought forth nuanced menstruation-related communication patterns across stratified groups. While 17.63% urban girls felt comfortable asking a question regarding menstruation to a school teacher, only 12.68% and 3.39% tribal and rural girls, respectively, reported the same. Additionally, 35.95% tribal girls who asked a question to their family members faced avoidance, as compared to 17.14% rural and 28.72% urban girls; thus revealing a pattern of unequal communication experiences among girls. The experience of facing avoidance was found to be more among boys as compared to girls, with about 58.97% urban boys and approximately 77% tribal and rural boys reporting it. A sense of comfort in asking a question to a school teacher was also found to be the highest among students admitted in private unaided schools (22.77% of girls and 42.55% of boys). About 53% sons of highly educated mothers as compared to 80% sons of uneducated mothers reportedly faced avoidance, indicating an unequal communication pattern among those whose mother is highly educated versus otherwise.

#### Social patterning in communication (Tables 3 and 4)

**Table 3:** Relationship of various social determinants with features of menstrual health communication: Prevalence ratios (95% Confidence Interval) (n = 744 boys and 677 girls).

	**Ever asked a question to family**			**Comfort to ask question to school teacher**			**Faced avoidance from family**		
** **	(1 = Yes)			(1 = Complete comfort)			(1 = Yes)		
	**Model 1**	**Model 2**	**Model 3**	**Model 1**	**Model 2**	**Model 3**	**Model 1**	**Model 2**	**Model 3**
Gender									
**Girls**	1	1	1	1	1	1	1	1	1
**Boys**	0.25 (0.21, 0.30)***	0.26 (0.16, 0.41)***	0.25 (0.15, 0.40)***	2.25 (1.80, 2.81)***	2.19 (1.75, 2.75)***	2.38 (1.80, 3.15)***	2.41 (1.99, 2.91)***	2.26 (1.83, 2.80)***	2.75 (2.04, 3.71)***
Setting									
**Rural**	1	1	1	1	1	1	1	1	1
**Tribal**	1.43 (1.18, 1.73)***	1.45 (1.12, 1.88)**	1.58 (1.15, 2.18)**	1.54 (1.09, 2.16)*	1.40 (0.88, 2.22)	1.10 (0.55, 2.18)	1.60 (1.12, 2.28)**	1.08 (0.70, 1.68)	1.51 (0.73, 3.09)
**Urban**	0.84 (0.69, 1.02)***	0.92 (0.76, 1.12)	0.82 (0.63, 1.07)	1.47 (1.06, 2.04)*	1.12 (0.80, 1.58)	1.22 (0.88, 1.69)	1.14 (0.78, 1.65)	0.91 (0.59, 1.39)	1.29 (0.76, 2.16)
Mother's Education									
**No/Some elementary schooling**	1	1	1	1	1	1	1	1	1
**Some middle school**	0.85 (0.73, 1.00)	1.16 (1.03, 1.31)*	1.23 (1.07, 1.42)**	0.85 (0.66, 1.11)	0.85 (0.61, 1.17)	0.92 (0.73, 1.16)	0.77 (0.59, 1.00)	0.85 (0.65, 1.12)	0.84 (0.64, 1.10)
**Completed high-school/Some college**	0.72 (0.58, 0.90)**	1.04 (0.81, 1.33)	0.96 (0.72, 1.29)	0.88 (0.64, 1.21)	0.88 (0.59, 1.30)	0.74 (0.49, 1.10)	0.71 (0.49, 1.03)	0.75 (0.49, 1.14)	0.99 (0.66, 1.48)
**Completed college or more**	0.80 (0.64, 1.00)	1.10 (0.83, 1.45)	0.86 (0.63, 1.17)	1.52 (1.17, 1.98)	1.36 (0.94, 1.97)	1.04 (0.70, 1.56)	0.73 (0.50, 1.08)	0.67 (0.42, 1.06)	0.92 (0.49, 1.71)
Father's Education									
**No/Some elementary schooling**	1	1	1	1	1	1	1	1	1
**Some middle school**	0.79 (0.67, 0.94)**	1.03 (0.86, 1.25)	1.04 (0.87, 1.25)	0.77 (0.58, 1.03)	0.81 (0.59, 1.12)	1.04 (0.79, 1.37)	0.92 (0.71, 1.20)	1.02 (0.85, 1.21)	1.03 (0.81, 1.30)
**Completed high-school/Some college**	0.66 (0.54, 0.81)***	0.95 (0.78, 1.15)	0.89 (0.66, 1.19)	0.87 (0.64, 1.19)	0.94 (0.65, 1.37)	1.28 (0.84, 1.94)	0.88 (0.64, 1.22)	0.96 (0.73, 1.26)	0.94 (0.63, 1.41)
**Completed college or more**	0.76 (0.63, 0.92)**	1.13 (0.80, 1.59)	1.21 (0.83, 1.76)	1.34 (1.03, 1.76)*	1.33 (0.92, 1.93)	1.46 (0.91, 2.33)	0.64 (0.45, 0.92)*	0.60 (0.40, 0.90)*	0.51 (0.26, 1.01)
Wealth tertile									
**Poorest**	1	1	1	1	1	1	1	1	1
**Middle**	0.71 (0.60, 0.85)***	0.98 (0.85, 1.13)	0.78 (0.69, 0.89)**	0.73 (0.55, 0.96)*	0.77 (0.54, 1.09)	0.76 (0.50, 1.16)	0.81 (0.61, 1.08)	1.09 (0.86, 1.37)	1.13 (0.83, 1.53)
**Richest**	0.75 (0.63, 0.89)**	1.17 (0.93, 1.48)	0.94 (0.77, 1.16)	1.17 (0.93, 1.49)	1.09 (0.75, 1.59)	0.90 (0.53, 1.54)	0.72 (0.54, 0.98)*	0.94 (0.58, 1.50)	1.22 (0.73, 2.02)
School type									
**Government**				1	1	1			
**Private aided**				0.74 (0.58, 0.93)*	0.91 (0.61, 1.34)	0.76 (0.37, 1.57)			
**Private unaided**				1.45 (1.13, 1.85)**	1.68 (1.09, 2.61)*	1.16 (0.49, 2.77)			

Statistically significant p values indicated as *<0.05, **0.01, ***<0.001; Model 1: Unadjusted, Model 2: Adjusted for Age, Setting and Gender, Model 3: Adjusted for School-Type and/or Parental Socioeconomic Factors

**Table 4:** The relationship of various social determinants with taboos being communicated to adolescent girls (Odds Ratios at 95% Confidence Interval) (n = 677).

** **	**No/Mild**	**Moderate**	**Severe**	**No/Mild**	**Moderate**	**Severe**	**No/Mild**	**Moderate**	**Severe**
** **	Model 1			Model 2			Model 3		
Setting									
**Rural**	1	1	1	1	1	1	1	1	1
**Tribal**	1	0.41 (0.22, 0.73)**	0.24 (0.15, 0.39)***	1	0.36 (0.19, 0.67)**	0.22 (0.14, 0.37)***	1	0.18 (0.09, 0.36)***	0.18 (0.09, 0.36)***
**Urban**	1	1.59 (1.06, 2.36)*	1.59 (1.06, 2.36)*	1	1.60 (1.06, 2.42)*	1.60 (1.06, 2.42)*	1	1.62 (0.94, 2.79)*	1.62 (0.94, 2.79)
Mother's Education									
**No/Some elementary schooling**	1	1	1	1	1	1	1	1	1
**Some middle school**	1	0.64 (0.45, 0.92)	1.54 (1.07, 2.21)*	1	0.71 (0.46, 1.11)	0.71 (0.46, 1.11)	1	0.65 (0.37, 1.14)	0.65 (0.37, 1.14)
**Completed high-school/Some college**	1	0.47 (0.30, 0.72)	2.12 (1.37, 3.28)**	1	0.88 (0.52, 1.48)	0.88 (0.52, 1.48)	1	0.63 (0.31, 1.26)	0.63 (0.31, 1.26)
**No/Some elementary schooling**	1	0.26 (0.16, 0.41)	3.77 (2.41, 5.92)***	1	1.25 (0.72, 2.17)	1.25 (0.72, 2.17)	1	0.64 (0.29, 1.41)	0.64 (0.29, 1.41)
Some Middle School									
**No/Some elementary schooling**	1	1	1	1	1	1	1	1	1
**Some middle school**	1	1.19 (0.79, 1.78)	1.19 (0.79, 1.78)	1	0.69 (0.43, 1.10)	0.69 (0.43, 1.10)	1	0.64 (0.36, 1.13)	0.64 (0.36, 1.13)
**Completed High-school/Some college**	1	2.12 (1.36, 3.30)**	2.12 (1.36, 3.30)**	1	0.94 (0.55, 1.59)	0.94 (0.55, 1.59)	1	0.91 (0.46, 1.80)	0.91 (0.46, 1.80)
**No/Some elementary schooling**	1	3.39 (2.20, 5.21)***	3.39 (2.20, 5.21)***	1	1.22 (0.70, 2.09)	1.22 (0.70, 2.09)	1	1.07 (0.50, 2.27)	1.07 (0.50, 2.27)
Wealth tertile									
**Poorest**	1	1	1	1	1	1	1	1	1
**Middle**	1	1.80 (1.21, 2.66)**	1.80 (1.21, 2.66)**	1	0.82 (0.50, 1.34)	0.82 (0.50, 1.34)	1	0.73 (0.41, 1.31)	0.73 (0.41, 1.31)
**Richest**	1	3.92 (2.66, 5.79)***	3.92 (2.66, 5.79)***	1	1.25 (0.72, 2.16)	1.25 (0.72, 2.16)	1	0.91 (0.45, 1.86)	0.91 (0.45, 1.86)

statistically significant p values indicated as *<0.05, **0.01, ***<0.001; Model 1: Unadjusted, Model 2: Adjusted for Age, Setting and Gender, Model 3: Adjusted for School-Type and/or Parental Socioeconomic Factors

Although fewer boys than girls reported asking a question [IRR at 95% CI: 0.25 (0.15, 0.40], more boys than girls faced avoidance [IRR at 95%CI: 2.75 (2.04, 3.71)]. Comfort with teacher was more among boys as compared to girls [IRR at 95% CI: 2.38 (1.80, 3.15)].

More tribal than rural adolescents had asked a question (IRR at 95% CI: 1.58 (1.15, 2.18)). Fewer girls from tribal than the rural area were communicated severe taboos (OR at 95% CI: 0.18 (0.09, 0.36)) and more urban versus rural girls were communicated moderate taboos (OR at 95% CI: 1.62 (0.94, 2.79)).

Adolescents with mothers having at least some secondary schooling had higher prevalence of asking a question than those with mothers having no or some primary schooling [IRR: 1.23 (1.07, 1.42)]. Fewer teens from middle than the poorest tertile asked a question (IRR at 95% CI: 0.78 (0.69, 0.89)), as per model 3.

### Influence of IP communication on health and quality of life (Tables 5 and 6)

**Table 5:** Relationship between communication-related factors and menstrual health-related outcomes (n = 744 boys and 677 girls).

** **	**Knowledge**			**Beliefs**		
** **	(1 = Possess)			(1 = Positive beliefs)		
** **	Model 1	Model 2	Model 3	Model 1	Model 2	Model 3
Ever asked a question						
**No**	1	1	1	1	1	1
**Yes**	1.92 (1.59, 2.33)***	1.27 (1.02, 1.58)*	1.19 (0.96, 1.49)	1.12 (0.88, 1.42)	1.78 (1.35, 2.35)***	1.77 (1.29, 2.43)***
Faced avoidance to questions						
**No**	1	1	1	1	1	1
**Yes**	0.53 (0.40, 0.72)***	0.59 (0.42, 0.84)**	0.59 (0.37, 0.94)*	0.78 (0.52, 1.15)	0.49 (0.33, 0.73)**	0.45 (0.23, 0.88)*
Comfort with teacher						
**Lack of complete comfort**	1	1	1	1	1	1
**Complete comfort**	1.25 (1.01, 1.55)*	1.56 (1.20, 2.02)**	1.41 (1.06, 1.88)*	1.72 (1.24, 2.39)**	1.72 (1.24, 2.39)**	1.72 (1.19, 2.49)**

statistically significant p values indicated as *<0.05, **0.01, ***<0.001; Taboos communicated, Stress regarding stain, Menstrual health status and Missed school during periods variables apply only for the adolescent girls sample (n = 677); Model 1: Unadjusted, Model 2: Adjusted for Age, Setting and Gender, Model 3: Adjusted for School-Type and/or Parental Socioeconomic Factors

**Table 6:** Relationship between taboos being communicated and menstrual health-related outcomes (n = 744 boys and 677 girls).

	Knowledge			Beliefs			Stress regarding stain			Missed school during periods			Menstrual health status								
	(1 = Possess)			(1 = Positive beliefs)			(1 = Yes)			(1 = Yes)			Poor			Moderate			Good		
	Prevalence Ratios (95% Confidence Interval)												Odds Ratios (95% Confidence interval)								
Taboos Being Communicated	Model 1	Model 2	Model 3	Model 1	Model 2	Model 3	Model 1	Model 2	Model 3	Model 1	Model 2	Model 3	Model 1	Model 2	Model 3	Model 1	Model 2	Model 3	Model 1	Model 2	Model 3
**No/Mild**	1	1	1	1	1	1	1	1	1	1	1	1	1	1	1	1	1	1	1	1	
**Moderate**	0.89 (0.67, 1.19)	0.92 (0.69, 1.23)	0.98 (0.74, 1.30)	0.51 (0.30, 0.87)*	0.50 (0.30, 0.85)*	0.46 (0.28, 0.75)**	1.20 (0.99, 1.45)	1.23 (1.01, 1.49)*	1.18 (0.95, 1.47)	1.00 (0.67, 1.49)	0.99 (0.66, 1.47)	1.16 (0.67, 1.99)	1	1	1	1	1.00 (0.58, 1.70)	0.80 (0.44, 1.47)	1	0.57 (0.26, 1.21)	0.50 (0.14, 1.79)
**Severe**	0.84 (0.65, 1.10)	0.84 (0.64, 1.10)	0.84 (0.60, 1.17)	0.51 (0.31, 0.82)**	0.50 (0.31, 0.81)**	0.53 (0.34, 0.83)**	1.31 (1.10, 1.56)**	1.43 (1.19, 1.72)***	1.31 (1.10, 1.57)**	1.18 (0.82, 1.69)	1.32 (0.90, 1.94)	1.69 (1.10, 2.59)*	1	1	1	1	1.12 (0.65, 1.92)	0.65 (0.34, 1.23)	1	0.63 (0.28, 1.41)	0.74 (0.39, 1.39)

statistically significant p values indicated as *<0.05, **0.01, ***<0.001; Taboos communicated, Stress regarding stain, Menstrual health status and Missed school during periods variables apply only for the adolescent girls sample (n = 677); Model 1: Unadjusted, Model 2: Adjusted for Age, Setting and Gender, Model 3: Adjusted for School-Type and/or Parental Socioeconomic Factors

Ever asking a question was associated with having positive beliefs (PR: 1.77 (1.29, 2.43)), whereas facing avoidance was related to fewer reports of positive beliefs (IRR: 0.45 (0.23, 0.88)) and knowledge [PR: 0.59 (0.37, 0.94)]. Comfort with teacher was associated with possessing knowledge (IRR: 1.41 (1.06, 1.88)) and positive beliefs [PR: 1.72 (1.19, 2.49)].

Girls who had been communicated moderate or severe taboos than those who had been communicated no/mild taboos had a lower adjusted prevalence of positive beliefs (IRR: 0.46 (0.28, 0.75) and 0.53 (0.34, 0.83), respectively). More girls who had been communicated severe taboos than those who had been communicated no or mild taboos had stress regarding stain (IRR: 1.31 (1.10, 1.57)) and missing school (IRR: 1.69 (1.10, 2.59)). The influence of taboo communication on missing school remained consistent and significant even after adjusting for girl’s menstrual health status.

#### Post-survey phase

Key respondents agreed with our main findings and expressed a need to understand the nuanced manner in which each setting affected the outcome-

“*Girls in urban slums*, *rural or tribal areas might all be staying in a small 10 by 12 [square feet] houses but whether they have a separate place to dry their menstrual clothes*, *whether their toilet is inside*, *whether they have a regular water supply to clean themselves when necessary and whether their menstruation remains a private matter… all such factors will determine the way they talk or not talk about menstruation and [will determine] whether they are being communicated taboos*.*”*

Newly married adolescent girls shared how they felt uncomfortable discussing anything related to menstruation with their husbands, which left them feeling lonely during periods. Interviews of school-dropout girls reflected their need for receiving information on the topic and for discussing their concerns as these needs seemed to remain unfulfilled due to a lack of institutional support of schools. Additionally, in order to triangulate our survey findings, we shared our survey results with the key respondents and adolescent participants in the post-survey qualitative study. An FGD with school dropout girls was conducted to seek their inputs on our findings, specifically to understand the barriers they faced in menstrual health communication due to the absence of school as a source of information and as a social environment. Additionally, key respondents were requested to provide feedback on our findings and provide any inputs to interpret our results. They were particularly requested to draw from their expertise and experience of working with boys, and disadvantaged adolescents to help us understand how communication inequalities emerge and influence menstruation-related outcomes. The resistance to discuss menstruation among boys despite being curious about the topic; absence of favorable beliefs among those who faced silence or menstrual taboos; were apparent in our survey data as well as in our field observations and interactions with adolescents, which further helped to triangulate our findings.

## Discussion

Overall, our study of 1421 adolescents from Nashik district from Western India showed that both adults and adolescents create a discouraging environment to discuss menstruation. While girls are communicated taboos and norms, both boys and girls face resistance against discussing menstruation, which adversely impacts their health and life. Our qualitative data showcased nuances of communication taboo and inequality faced by adolescents in terms of the access to information, responses to communication, whereas, our quantitative data brought forth the patterns in this inequality not just between adolescents from different backgrounds and gender but also within each subgroup and its measurable health impact.

Our research corroborates previous findings among Indian adolescents which suggest that the gender is important in determining the quality, content, and nature of SRH communication [5,16,35,36]. Although few studies emphasize the disadvantages of poor socioeconomic setting on girls’ menstrual hygiene experiences, our study contributes by further highlighting the ways in which socioeconomic setting influences the menstruation-related communication experience of both genders [37,38]. Evidence suggested that feeling fearful of talking; being unable to communicate menstrual illness; facing avoidance; being communicated severe taboos, adversely affected adolescents’ health. We are unaware of any mixed-methods study from India or abroad which includes adolescents of both genders from diverse socioeconomic settings while studying pathways connecting social determinants of IP communication with menstrual health. However, our findings were similar to the pathways connecting determinants of broader SRH communication with adolescents’ health from India and abroad [10,16,20,39,40].

Our results suggest that despite being curious, boys face avoidance to their questions by family, potentially making them rely more on media or peers to seek answers [1,3,4]. Our finding suggesting more boys felt comfortable asking questions to their teacher was backed by the social worker we interviewed as he explained how poor families largely remain busy in earning their daily wages, with no time to attend to the SRH needs of their children. Additionally, she explained, unlike boys, girls get a window to discuss their lived experience in homes, making schools an important place for boys to learn about SRH. However, the impact of school environment in creating comfort among students is not well explored in Indian literature.

Our qualitative data also throws light upon the differences in communication-related experiences among adolescents. Gender and socioeconomic disparities in menstruation-related communication experiences affected adolescents’ social construction of menstruation as well as their knowledge, beliefs and health outcomes. For instance, girls from a disadvantaged background reported experiencing shame as they tried to hide their menstruation-related complaints from their parents and further avoided seeking healthcare; whereas wealthy urban girls were found to be more open about their menstruation to their parents. Differences in communication were, however, also found within specific socioeconomic categories, highlighting the role of gender in driving menstruation-related communication. For instance, while a highly educated urban mother was more willing to open up about menstruation to her daughter, the mother of an urban boy from a similar socioeconomic background was found to justify her avoidance in talking to her son. Communication-related differences were also found to be shaped by parents’ availability, awareness, and interest in talking about sensitive topics, some of which were rooted in their social status and economic circumstance. For example, at least three wealthy urban girls reported menstruation-related communication with their fathers. This experience was found to be lacking among rural girls, while tribal parents reportedly struggled to keep their daughters in schools due to financial hardship and due to the societal burden of marrying girls at an early age, leaving no scope for parent-daughter communication on any sensitive topic.

Our study supports previous research showing that positive IP communication is beneficial for SRH outcomes [5,17]. We highlighted how taboos being communicated affect girls’ health (stress regarding stain) and quality of life (school absenteeism). Although a study showed that girls facing restrictions on sports and exercise had higher odds of [OR: 8.7 (6.20, 12.4)] missing school [13], our study contributes further by showing the negative impact of various taboos on girls’ health, even after adjusting for menstrual health status; an important finding pointing towards how girls embody taboos being communicated [41].

Our qualitative findings suggest that emphasis needs to be given to the specific interpersonal communication-related needs of adolescents (such as creating an enabling environment for girls to discuss menstrual health with the community-based health practitioners) and responsible adults (such as, equipping teachers with menstrual health education tools and resources) while designing community- and school-level policies focusing on menstrual health. This could be done using creative and interactive ways such as drama, where information could be combined with ways to develop their emotional intelligence and gender sensitivity Moving beyond information dissemination, policies needs to shift towards creating healthy platforms for adolescents to discuss sensitive topics [42]. The urgent need to equip responsible adults with appropriate communication skills is clear from our qualitative findings. Our key informant interviews suggested a clear need for parents support groups and gender sensitization training, especially for male teachers, in order to break taboos and build the confidence to talk about sensitive topics such as menstruation. Our quantitative findings suggest that the specific requirements of adolescents from different socioeconomic settings could be addressed by looking into the complexities of structural and social attributes of different settings. The necessity of such an approach was evident as we were able to see several girls, especially from poor urban and rural setting, facing pre-menarcheal silence; while boys from non-urban settings resisted opening up about their menstruation-related experiences and curiosity. Our interviews with key respondents suggested that theatre techniques and innovative gender sensitizing games, in addition to openly discussing menstruation, would be an essential step in improving IP communication across different socioeconomic settings. However, equipping teachers, especially the men in rural and tribal settings, with resources and tools to boost their confidence in discussing this sensitive topic seems an urgent need. Health practitioners across settings and disciplines could be urged to educate the adolescent girls and their parents so that girls feel at ease sharing their health problems. Our data also suggested the need for appointing a visiting adolescent psychologist in all schools who could help girls facing negative beliefs regain their confidence and self-body image as they go through the transition phase of adolescence. Especially in the tribal setting, school education policies could be expanded to specifically address the societal pressure faced by parents to get their daughters married early.

### Limitations

Non-probabilistic sampling design is a limitation; however, our sample was large enough to find patterns and nuances of menstrual health communication through a mixed-methods approach, a topic not studied before. We could not incorporate adolescents between ages 10 to 12 years and school-dropouts thus limiting our ability to generalize to a wider population. Knowledge and beliefs were operationalized as simple additive composite measures that lack to capture the intricacies of these constructs.

Although previous evidence has shown that mother’s education and parental income affect adolescents’ SRH-related behaviors and outcomes, our study did not find evidence of their effect through menstruation-related IP communication [2]. As parental socioeconomic data were reported by adolescents, it may have limitations due to inaccurate information, and personal aspirations. These data were not cross-examined through school records; hence, accuracy of the data could not be verified. Similarly, the wealth data had more than 10% missing values. A comparison with the figures for Nashik district from a representative survey indicated a lack of representation of the poorest two categories of household wealth in our data, which may explain the inconsistent and weak patterns with wealth in our study [43]. The non-representation of the poorest two categories of the population could partially be explained by our school-based sampling strategy which could not capture school-dropouts, who likely belonged to the poorest groups. Despite the noise in data, we were able to detect that mother’s secondary school-level education (than having no/some primary schooling) increased the chances of asking a question to family members.

### Strengths

Our study has several strengths. Firstly, a mixed methods approach allowed us to interpret the data coherently and our thick description added value by highlighting the nonverbal cues and silence, which often remain unaddressed in academia. Secondly, an inclusive approach towards gender and socioeconomic setting showed similarities and differences pertaining to adolescents’ different structural location and social position, giving rise to communication inequalities that are often neglected by policymakers. Moreover, our study underscores that these different social identities and structural locations interconnect giving rise to complex social realities for adolescents. Thus, we highlight the need to complicate the concept of communication inequality by applying the lens of intersectionality.

## Conclusion

In order to increase effectiveness and inclusivity while communicating with adolescents regarding sensitive topics, public health campaigns should widen their approach from health information communication to a more nuanced communication-strategy that addresses the roots of communication inequality and communication taboos around sensitive topics. Using young men as community peer leaders to conduct gender-focussed workshops and discussions; implementing a social and behavioral change communication strategy that uses infotainment tools and involves health workers in the public health system in facilitating interpersonal communication on sexual and reproductive health; or designing a comprehensive school-based health education program have shown significant improvement in adolescent/ youth’s perceptions regarding various taboo topics such as sexual and reproductive health [17,44,45]. Such interventions, in addition to their focus on improving SRH outcomes, could make the inclusion of marginalized adolescents an explicit aim. Their implementation would thus include those whose disadvantaged backgrounds deprive them of the spaces and opportunities to discuss menstruation at home and in schools. An inclusive approach for gender and socioeconomic settings can pave a way for designing a comprehensive policy that addresses the specific needs of adolescents at different intersections of society.

## Supporting information

S1 Checklist Appendix B_COREQ-32-item checklist. (DOCX)

S1 Interview Guide Appendix A_Semi-structured interview guide. (DOCX)

S1 Document Appendix C_Variable operationalization. (DOCX)

S2 Document Appendix D_Questions used for analysis. (DOCX)
