# Supplementary material for: Menstrual health communication among Indian adolescents: A mixed-methods study
Source: PLoS One. 2019 Oct 17;14(10):e0223923. doi: 10.1371/journal.pone.0223923 (PMC6797238; doi:10.1371/journal.pone.0223923)
Supplement: S1 Checklist — (DOCX) [file pone.0223923.s001.docx]

| COREQ-32 ITEM CHECKLIST FOR QUALITATIVE PHASE | | | |
| --- | --- | --- | --- |
| **No** | **Item** | **Guide questions/description** | **Responses** |
| **Domain 1: Research team and reflexivity** |  |  |  |
| Personal Characteristics |  |  |  |
| 1. | Interviewer/facilitator | Which author/s conducted the interview or focus group? | Gundi, M. and Research assistant (male) |
| 2. | Credentials | What were the researcher's credentials? *E.g. PhD, MD* | At the time of data collection, Gundi, M. was a PhD student at IIT Gandhinagar with a Master of Public Health degree and the male research assistant was a freelance social science researcher with a master's degree in journalism. |
| 3. | Occupation | **Simply state in the author contributions section that the data was collected as part of your doctoral work.** | The data was collected as a part of Gundi, M.'s doctoral dissertation work. |
| 4. | Gender | Was the researcher male or female? | Gundi, M. is a female and research assistant is a male |
| 5. | Experience and training | What experience or training did the researcher have? | At the time of data collection, Gundi, M. had about two years of experience of working in urban, rural and tribal communities in Maharashtra. She had previously conducted semi-structured interviews and focus group discussions of women, and girls regarding sensitive topics, including that of menstruation. The research assistant had previous experience of working in disadvantaged communities including those in the Nashik district, in India. He had previously conducted semi-structured interviews as well as focus group discussions of community members on various topics related to social sciences, including those of health. The researcher had taken the courses on research methods, community health, public health foundation as a part of her master's and a PhD degree. Some of these courses also required desigining a questionnaire, collecting data in the field and developing training modules. Male data collector too had done a course on research methods, communication and and internship in health communication as a part of his master's degree. Prior to commencing this study, researchers insured that the research assistant has a sound understanding of the research questions and the broad areas that need to be captured during the qualitative data collection. Two mock interviews had been conducted prior to data collection. |
| Relationship with participants |  |  |  |
| 6. | Relationship established | Was a relationship established prior to study commencement? | Prior to commencing the study, researcher had made one visit to a remote school in tribal setting, and two schools in the urban area. The researcher ensured to meet school teachers in advance, in order to gain insights about the procedures and requirements regarding obtaining consent, and carrying out the study among adolescents. Researcher had also met few tribal and urban adolescents as a part if this field visit to gauge their interest and perceived importance to participate in a study focusing on this sensitive topic |
| 7. | Participant knowledge of the interviewer | What did the participants know about the researcher? e*.g. personal goals, reasons for doing the research* | Prior to collecting the data among all the settings, researchers ensured to introduce themselves to the school teachers, participants, other concerned authorities and provided a detailed information regarding who they were; which geographical area they belong to, which educational institutions they belonged to and the motivation for this study. They explained in detail the goal for this study topic as 'a research study that investigates how adolescent girls and boys from urban, rural and tribal areas understand and think about menstruation, what their experiences and concerns are." and their rationale for conducting this study. They also provided a detailed information about the steps and phases in this study, and the ethical approvals received. All the participants were provided the opportunity to ask any questions or concerns that they might had. Only after receving consent from parents, schools gave us permission to conduct this study. A detailed account of all of this information was included as a part of the informed consent process prior to collecting the data. |
| 8. | Interviewer characteristics | What characteristics were reported about the interviewer/facilitator? e.g. *Bias, assumptions, reasons and interests in the research topic* |  |
| **Domain 2: study design** |  |  |  |
| Theoretical framework |  |  |  |
| 9. | Methodological orientation and Theory | What methodological orientation was stated to underpin the study? *e.g. grounded theory, discourse analysis, ethnography, phenomenology, content analysis* | Social constructionist approach along with a framework of social determinants of health helped us draw the conceptual model of this study prior to data collection. This conceptual model guided the collection, analysis, and presentation of qualitative (and the quantitative) data in this study. An iterative process of data collection and analysis-- that allowed girls' and boys' nuanced observations, experiences and thoughts regarding menstruation to be captured to guide the two phases of qualitative data collection, coding, as well as the thematic analysis, as described by Creswell et al. (2003) and Braun et al. (2019). |
| Participant selection |  |  |  |
| 10. | Sampling | How were participants selected? *e.g. purposive, convenience, consecutive, snowball* | A school teacher from an urban private aided school, the head of the local NGO working with adolescents and youth for media and communication for development and a sex-education expert helped identify and recruit participants face-to-face for the interviews and FGDs in both the qualitative phases of the study. Adolescents and key respondents who were interested in and agreed to participate in qualitative data collection, provided verbal consent and provided basic demographic and contact information apart from answering to menstruation-related questions. There were total 42 semi-structured interviews of adolescents (21 girls+21 boys), 12 key respondents' interviews and 9 focus group discussions in the first qualitative data collection phase. In the second phase, which was conducted post the cross-sectional survey, there were 5 semi-structured interviews of girls, 1 FGD (school dropout girls) and 3 key respodents' interviews. Among all the participants, only one adolescent boy from an urban setting expressed desire to quit the focus group discussion (as he felt uncomfortable to discuss this topic), who was also referred to an adolescent psychologist to ensure his emotional wellbeing. Since this study focused primarily on menstruation, participants of both genders and varied socioeconomic settings, were selected to represent a broad range menstruation-related experiences that could potentially be affected by their setting, and socioeconomic background. To achieve this representation and inclusion of participants from varied backgrounds, we also took assistance from school teachers to ensure that only those students who perform well in the academics are not chosen as a bias. Although all the interviews were recorded, few interviews were not recorded to respect participants' reservations for their opinions being audio-recorded. Some participants also skipped fee questions if they felt uncomfortable answering those questions. |
| 11. | Method of approach | How were participants approached? e*.g. face-to-face, telephone, mail, email* |  |
| 12. | Sample size | How many participants were in the study? |  |
| 13. | Non-participation | How many people refused to participate or dropped out? Reasons? |  |
| Setting |  |  |  |
| 14. | Setting of data collection | Where was the data collected? e*.g. home, clinic, workplace* | Semi-structured interviews and focus-group discussions took place either at participants' homes, schools or workspaces, in a space that was quiet, comfortable and ensured participants' privacy. In the rare event, one FGD was conducted at a local non-governmental organization working on sanitation. No person other than the participant/s and the interviewer was present during data collection. In a rare event that someone entered the space, interviewers waited for that person to leave before recommencing the interview. |
| 15. | Presence of non-participants | Was anyone else present besides the participants and researchers? |  |
| 16. | Description of sample | What are the important characteristics of the sample? *e.g. demographic data, date* | Since this study focused on adolescents' understanding and experiences regarding menstruation, adolescents were iteratively selected from different socioeconomic settings and from different socioeconomic backgrounds within each setting. This was to capture diverse experiences regarding menstruation among boys and girls from urban, rural and tribal areas, parents' ability to offer educational opportunities to their children; as per our hypothesis and conceptual framework. In addition to this, we also tried to include parents/guardians of both genders from different socioeconomic settings. Within urban setting, we conducted interviews and FGDs in schools located in high as well as low resource setting areas (urban slums) to capture the diversity within urban space. Healthcare providers practicing with different expertise and philosophies (Allopathic, Ayurvedic, Homeopathic and Psychologist) were interviewed to understand how menstrual illnesses and ways to address them differ. In phase 2 of the qualitative data collection, we also included school-dropout adolescent girls, and married adolescent girls, to capture their unique experiences regarding this topic. |
| Data collection |  |  |  |
| 17. | Interview guide | Were questions, prompts, guides provided by the authors? Was it pilot tested? | Interview guides, possible probes, and potential questions were developed by the authors and reviewed by a public health scholar, an adolescent sex-education expert and a school teacher. Two additional professors from IIT Gandhinagar including all authors of this study and the members of the institutional ethics committee reviewed this guide. Based on our initial few interviews, we modified the interview guide for appropriate wording and effective ordering. |
| 18. | Repeat interviews | Were repeat interviews carried out? If yes, how many? | No repeat interviews were carried out. |
| 19. | Audio/visual recording | Did the research use audio or visual recording to collect the data? | The researchers used audio recording to collect the data. All participants consented, separately, to the audio recording of the interviews. At any point if participant wanted to discuss something off-record, we respected it by switching off the recorder. Three boys' interviews and one focus group discussion was not recorded as they expressed nervousness to speak on-record, while, still wishing to participate in the study |
| 20. | Field notes | Were field notes made during and/or after the interview or focus group? | Field notes were taken both during and after the interviews by both the interviewers. The thick description and field notes helped in the interpreting and analyzing the qualitative data. |
| 21. | Duration | What was the duration of the interviews or focus group? | The interviews lasted from 25-45 minutes, while, the FGDs lasted from 50-80 minutes. |
| 22. | Data saturation | Was data saturation discussed? | We stopped collecting the data upon meeting saturation. We looked at the variety in menstruation-related experiences across different socioeconomic settings and among both genders. |
| 23. | Transcripts returned | Were transcripts returned to participants for comment and/or correction? | No, transcripts were not returned to participants for comment and/or correction. |
| **Domain 3: analysis and findings** |  |  |  |
| Data analysis |  |  |  |
| 24. | Number of data coders | How many data coders coded the data? | Two |
| 25. | Description of the coding tree | Did authors provide a description of the coding tree? | All adolescent boys and girls in the study were asked to describe their understanding, beliefs, various observations and experiences regarding menstruation and how they faced/did not face communication-related barriers, taboos. Adolescents were further probed to describe how they perceived silence around the topic, if they ever faced it. Girls were specifically asked about their menstrual absorbents, health and illness and treatment-related communication-experiences. The responses to these questions constituted majority of the data that was analyzed in this study. Each coder, had their own understanding, concepts that guided the coding procedure which also contributed in a deeper understanding of how menstruation-related communication was similar/different among different socioeconomic backgrounds; how certain individuals had unequal access to resources, attention, opportunities to discuss this topic. |
| 26. | Derivation of themes | Were themes identified in advance or derived from the data? | Some broad areas formed the basis for developing the interview guide. However, new themes that emerged during the data collection and also while coding the data were also identified and analyzed. |
| 27. | Software | What software, if applicable, was used to manage the data? | The thematic analysis of the qualitative data was done manually. |
| 28. | Participant checking | Did participants provide feedback on the findings? | In the phase 2 of the qualitative phase, along with the quantitative findings, main themes and insights from the qualitative phase 1 were shared with key respondents. They agreed with our findings and provided constructive feedback which helped in interpreting the data. |
| Reporting |  |  |  |
| 29. | Quotations presented | Were participant quotations presented to illustrate the themes / findings? Was each quotation identified? e*.g. participant number* | Yes, participants' quotes were used to illustrate the findings in the manuscript. To respect the anonymity of the participants, pseudonyms were used along with participants' long quotes. |
| 30. | Data and findings consistent | Was there consistency between the data presented and the findings? | Yes |
| 31. | Clarity of major themes | Were major themes clearly presented in the findings? | Yes |
| 32. | Clarity of minor themes | Is there a description of diverse cases or discussion of minor themes? | Yes |
