# Supplementary material for: Menstrual health communication among Indian adolescents: A mixed-methods study
Source: PLoS One. 2019 Oct 17;14(10):e0223923. doi: 10.1371/journal.pone.0223923 (PMC6797238; doi:10.1371/journal.pone.0223923)
Supplement: S1 Interview Guide — (DOCX) [file pone.0223923.s002.docx]

**APPENDIX B**

**SEMI-STRUCTURED INTERVIEW GUIDE**

**Note to reviewers:**

Kindly note that this interview guide was used for collecting qualitative data using semi-structured interviews from adolescents only after receiving verbal assent from participants’ parents and teachers and after receiving a verbal consent from adolescent participants.

Kindly also note that the socio-demographic information we sought is frequently asked in various settings (including their own school) and is not considered private or sensitive in the Indian context. Because these are familiar questions which they have likely answered earlier, it can help to begin interviews by collecting this information, to just get into the flow of the conversation. While obtaining the assent and consent, we had provided a detailed account of what will be asked in the interviews, including the questions pertaining to socio-demographic and questions specific to the topic of study.

**FOR ADOLESCENT GIRLS:**

**(Note to the interviewer:** Begin only after receiving the verbal consent from the participant. Please assure complete anonymity to the participant and start audio-recording only after receiving consent for the same. Sentences given below are just the suggested opening for the interview, after introducing yourself to the participant.**)**

*“Hello, how was your day? Would you like to tell us a little more about your family? We will then proceed to discuss what you know about your menstruation and what are your menstruation-related thoughts and experiences. If any of our questions make you uncomfortable, please feel free to not answer that question. Shall we begin?”*

“नमस्कार/हॅलो! तुझा आजचा दिवस कसा गेला? तुझ्या कुटुंबाबद्दल आम्हाला थोडी माहिती देशील का? ते सांगून झालं की आपण तुला मासिक पाळीबद्दल काय माहित आहे, तुझे पाळीविषयी काय विचार आहेत, काय अनुभव आहेत; याविषयी बोलू. जर यापैकी कुठल्याही प्रश्नाने तुला अस्वस्थ वाटले, जर एखादा प्रश्न तुला आवडला नाही, तर त्या प्रश्नाचं उत्तर तू दिलं नाहीस तरी हरकत नाही. आपण तुझी मुलाखत सुरू करूया का?”

**Date of the interview:**

**Place and time:**

**Name:**

**Age in years:**

**Name of the School:**

**Which standard in school:**

**Religion:**

**Caste:**

**Parental Education and occupation:**

Father’s education

Father’s occupation:

Mother’s education:

Mother’s occupation:

**Number of family members and relations:**

**Can you tell me something about your family and friends?**

**“तुझ्या कुटुंबाबद्दल, मित्र-मैत्रिणीबद्दल आम्हाला माहिती देशील का?”**

(Probes, such as: Members in the family, what do they do, who are you most attached to? Do you have siblings? Are they younger to you or elder to you? Who are your friends? How do you usually enjoy together? What do you chat about?)

**“Can you give me some information related to your house?”**

**“तुझ्या घराबद्दल काही माहिती आम्हाला जाणून घ्यायला आवडेल.”**

(Probes, such as: Where do you stay? How many rooms? Do you have a toilet? Is it inside, attached or outside the house? Do you have a separate bathroom? Have you always had a toilet or is it recently built? Which facilities available at home? Do you have a private room for yourself in the house? Do you have a dustbin in the house or in the toilet?)

**Do you know what menstruation is? Can you explain to me why and how does that occur?**

**तुला मासिक पाळी म्हणजे काय हे माहित आहे का? तू मला मासिक पाळीच्या दरम्यान काय होते, का होते हे सांगू शकशील का?**

**(Probes, such as:** does it occur among girls or boys or both? Do you know why it occurs? What happens during this process?**)**

**Has your periods started?**

**तुला कधी मासिक पाळी आली आहे का?**

**When did you have your first periods and how was the experience? What was told to you?**

**सर्वात पहिल्यांदा तुला पाळी कधी आली? तो अनुभव कसा होता? त्यावेळी तुला काय सांगितले गेले?**

(Probes, such as: Details of the first experience; did you know about periods before you got them? Who communicated to you about it? What was told to you about periods? Who told it? Who did you discuss your questions with? What was your siblings’ /friends’ reaction? Were any of your questions avoided/ silenced? Were any taboos communicated to you?)

**If any specific instructions regarding behavior and practices during menstruation were given, do you follow those instructions?**

पाळीच्या दिवसात कसे वागायचे, काय करायचे याविषयी काही विशिष्ट सूचना दिल्या असल्यास, त्या तू पाळतेस का?

(Probes, such as: If yes, how and which ones; if no, why not; what happens if you forget any of those instructions? Who reminds you about these instructions? Do you follow anything apart from what is being told to you? Were you anytime scolded, if you did not follow them?)

**Was anything regarding this topic discussed in school? Was this topic taught in your school?**

**या विषयावर शाळेत कुठलीही चर्चा झाली होती का? शाळेत हा विषय शिकवला गेला का?**

**(**Probes, such as: What did teacher teach about this topic? What was the gender of the teacher? Was there anything skipped from the syllabus? How do you find comfortable to discuss this topic with your teacher? Was anything discussed among school friends? Any sex-education workshop conducted in school?**)**

**Would you like to share if you have any health problems related to menstruation that you had in the past or now?**

**मासिक पाळीशी संबंधित कुठलाही त्रास तुला यापूर्वी झाला आहे का, किंवा आत्ता आहे का?**

(Probes, such as: Who do you share your problems with? Who takes decision of seeing a doctor when a problem is related to menstruation? Did you talk to the doctor about these menstruation-related health issues? What did doctor tell you?)

**Do you talk about menstruation related experience, problems, concerns, fears or information with your father, brother or male friends if you have any?**

**मासिक पाळीशी संबंधित अनुभव, प्रश्न, काळजी, भीती किंवा माहिती तू तुझ्या बाबांशी, भावाशी किंवा एखाद्या मित्राशी बोलतेस का?**

(Probes, such as: If no, why not? What was the comfort level? What was their reaction? Why should they know or not know?)

**Please feel free to ask me any questions that you might have:**

**तुला काही प्रश्न विचारायचे असल्यास जरूर विचार**

**Any observations and field notes from the interviewer:**

**Thank you very much for your valuable time and inputs!**

**FOR ADOLESCENT BOYS:**

**(Note to the interviewer:** Begin only after receiving the verbal consent from the participant. Please assure complete anonymity to the participant and start audio-recording only after receiving consent for the same. Sentences given below are just the suggested opening for the interview, after introducing yourself to the participant.**)**

*“Hello, how was your day? Would you like to tell us a little more about your family? We will then proceed to discuss what you know about your menstruation and what are your menstruation-related thoughts and experiences. If any of our questions make you uncomfortable, please feel free to not answer that question. Shall we begin?”*

“नमस्कार/हॅलो! तुझा आजचा दिवस कसा गेला? तुझ्या कुटुंबाबद्दल आम्हाला थोडी माहिती देशील का? ते सांगून झालं की आपण तुला मासिक पाळीबद्दल काय माहित आहे, तुझे पाळीविषयी काय विचार आहेत, काय अनुभव आहेत; याविषयी बोलू. जर यापैकी कुठल्याही प्रश्नाने तुला अस्वस्थ वाटले, जर एखादा प्रश्न तुला आवडला नाही, तर त्या प्रश्नाचं उत्तर तू दिलं नाहीस तरी हरकत नाही. आपण तुझी मुलाखत सुरू करूया का?”

**Date of the interview:**

**Place and time:**

**Name of the participant:**

**Age in years:**

**Name of the School:**

**Which standard in school:**

**Religion:**

**Caste:**

**Parental Education and occupation:**

Father’s education

Father’s occupation:

Mother’s education:

Mother’s occupation:

**Number of family members and relations:**

**Can you tell me something about your family and friends?**

“तुझ्या कुटुंबाबद्दल, मित्र-मैत्रिणीबद्दल आम्हाला माहिती देशील का?”

(Probes, such as: Members in the family, what do they do, who are you most attached to? Do you have siblings? Are they younger to you or elder to you? Who are your friends? How do you usually enjoy together? What do you chat about?)

**“Can you give me some information related to your house?”**

“तुझ्या घराबद्दल काही माहिती आम्हाला जाणून घ्यायला आवडेल.”

(Probes, such as: Where do you stay? How many rooms? Which facilities available at home? Do you have a private room for yourself in the house?)

**Do you know what menstruation is? Can you explain to me why and how does that occur?**

**तुला मासिक पाळी म्हणजे काय हे माहित आहे का? तू मला मासिक पाळीच्या दरम्यान काय होते, का होते हे सांगू शकशील का?**

**(Probes, such as:** does it occur among girls or boys or both? Do you know why it occurs? What happens during this process?**)**

**Have you ever tried asking anything about this topic to family members? Can you tell me what happened if you did so?**

**या विषयावर तू कुटुंबातील कुणाला कधी काही प्रश्न विचारले आहेत का? विचारलेस असशील तर, विचारल्यावर नेमके काय झाले ते सांगशील का?**

**(Probes, such as:** Were you given detailed answers to your questions? Were you able to ask your questions confidently? How did you feel when you received answers or did not receive answers? What was the reason given for denying answers? What did you do when you received information or were denied information?**)**

**Have you ever tried to speak about this topic with a male/female school teacher? What was your experience like?**

**या विषयावर तू कधी शाळेतील शिक्षकांशी/शिक्षिकेशी चर्चा केली आहेस का? तुझा अनुभव कसा होता?**

**Have you discussed this topic or anything related to this topic with your male/female friends?**

**या विषयावर / या विषयाशी संबंधित) तू तुझ्या मित्र अथवा मैत्रिणींबरोबर काही बोलला आहेस का?**

**(Probes, such as:** what did you discuss? Who brought up the topic? Were you talking comfortably?**)**

**Please feel free to ask me any questions that you might have:**

**तुला काही प्रश्न विचारायचे असल्यास जरूर विचार**

**Any observations and field notes from the interviewer:**

**Thank you very much for your valuable time and inputs!**
