# Supplementary material for: Menstrual health communication among Indian adolescents: A mixed-methods study
Source: PLoS One. 2019 Oct 17;14(10):e0223923. doi: 10.1371/journal.pone.0223923 (PMC6797238; doi:10.1371/journal.pone.0223923)
Supplement: S1 Document — (DOCX) [file pone.0223923.s003.docx]

**Appendix A: Details regarding Variables of Interest**

*Predictors:*

- School setting was based on the location of the school (rural/ tribal/ urban) and type was based on the funding source for the school (government, private aided, private unaided).
- Mother’s and father’s educational status were categorized as no/some elementary schooling, middle school, high-school/some college and completed college or more.
- We computed the household wealth score by using the first component of a Principal Component Analysis using 19 variables indicating possession of household assets and it was operationalized as a categorical variable with tertiles (poorest, middle, and richest).
- Whether information on menstruation was received (received information in schools) in schools was measured as yes or no. Adolescents’ perceived comfort with teachers to ask menstruation-related questions (comfort with teachers) had three categories, viz., not at all comfortable, comfort dependent upon the gender of the teacher, and completely comfortable irrespective of the gender of the teacher. Due to statistical limitations, we operationalized this as a binary variable (lack of complete comfort/ complete comfort).

*Covariates:*

Age (early/late adolescents); caste [general (socially privileged caste-group)/other]; religion (Hindu/non-Hindu); mother’s occupation (farmer, daily wage worker, employed, self-employed), and father’s occupation (farmer, daily wage worker, employed/self-employed, other) were used as covariates.

*Outcomes:*

*Communication-related outcomes:*

- Ever asking a question regarding menstruation to family members [asking a question] (asked a question/not) was one of the communication-related outcomes.
- Those who had ever asked a question were asked regarding facing avoidance by family member [facing avoidance] (faced avoidance/not).
- Comfort to ask question on menstruation was operationalized as [comfort with teacher] (complete comfort/lack of complete comfort).
- For girls, the extent of taboos being communicated was tested using taboo variable, an ordinal variable created based on whether girls were told to: follow religious restrictions, keep menstruation a secret from males in the family, sit away, not to touch, not talk to boys. These were later used as predictors to check their influence on health-related outcome variables.

*Health-related outcomes:*

- The composite variable ‘knowledge’ (possess/do not possess) was created by adding four mutually exclusive variables that tested whether participants possessed age-appropriate information on basic physiology of menstruation- such as ‘whether menstruation occurs among men or women’; ‘approximate age of menarche’, ‘reproductive age of women’, ‘whether menstrual blood flows out through vagina or urinary tract’.
- The composite variable ‘beliefs regarding menstruation’ (beliefs) (favorable, unfavorable) was an additive variable created on the basis of whether or not one believes that ‘menstruation is a curse for women’, ‘whether boys should know about menstruation or not’ and ‘whether women are dirty during menstruation’.
- The variable menstrual health status (MHS) was created by adding girls’ responses about various menstrual illness-related symptoms ever faced, such as: extreme pain during periods, itching near private parts, foul smell near groin, excess white discharge, periods multiple times in a month, periods missed, general weakness; to create an ordinal variable (poor MHS, moderate MHS, good MHS).
- Stress regarding stain of periods (yes/no) was measured among girls
- To test their quality of life, we measured if they ever missed school due to periods (yes/no).
