# Supplementary material for: Menstrual health communication among Indian adolescents: A mixed-methods study
Source: PLoS One. 2019 Oct 17;14(10):e0223923. doi: 10.1371/journal.pone.0223923 (PMC6797238; doi:10.1371/journal.pone.0223923)
Supplement: S2 Document — (DOCX) [file pone.0223923.s004.docx]

APPENDIX D

QUESTIONS FROM THE QUESTIONNAIRE USED FOR THE ANALYSIS

| **Note: Following questions from our questionnaire were used for the analysis of the quantitative data presented in this manuscript. The questionnaire for girls and boys were printed separately, in both English and Marathi languages, where participants were given a choice of the language. In the questions given below, the shaded cells represent questions that were asked specifically to adolescent girls (and were present only in girls' questionnaire), all other questions were common in both girls' and boys' questionnaires.** | | | |
| --- | --- | --- | --- |
| Hello! My name is (). I am a doctoral student of Public Health from (). I am conducting this survey to understand the health concerns of teenage girls and boys. If you fill this survey, it will help in my study which is important for my PhD. This survey is completely anonymous. Neither I nor my team will know your name and identity. This survey will not affect your school grades in any way. The information you share will not be shared with your teachers or anyone else in your school. You are free not to fill this survey as you have the freedom to not fill this up. This is a voluntary activity. If you have any questions, please feel free to contact me on (). Thank you for participating in this survey!  A sincere thank you! | | नमस्कार! माझे नाव (). मी (संस्थेचे नाव) येथे सामाजिक आरोग्य या विषयात पी.एच.डी. करते. तुम्हाला मासिक पाळीच्या आरोग्याविषयी काय माहितीआहे, समस्या काय आहेत, हे जाणून घेण्यासाठी मी हा सर्वे घेत आहे. हा सर्वे तुम्ही भरून दिलात तर माझ्या अभ्यासाला मदत होईल. या सर्वेमध्ये तुमची ओळख पूर्णपणे खाजगी राखली जाईल. यात तुमचे नावही विचारलेले नाही. या सर्वेचा तुमच्या शाळेतील गुणांवर मुळीच परिणाम होणार नाही. तुमच्या शिक्षकांना किंवा इतर कुणालाही तुम्ही दिलेली माहिती सांगितली जाणार नाही. हा सर्वे भरून देणे मुळीच बंधनकारक नाही.  तुम्हाला काही शंका असल्यास जरूर मला विचारा. माझा मोबाईल नंबर आहे- ()  सर्वेमध्ये सहभागी झाल्याबद्दल धन्यवाद! | |
| **Form number** |  | **फॉर्म क्रमांक** |  |
| **Date** |  | **तारीख** |  |
| **School name** |  | **शाळेचे नाव** |  |
| **Standard** |  | **इयत्ता** |  |
| **Locality of the residence/ Any landmark near your residence** |  | **घराजवळची खूण/ कोणत्या भागात राहता ते लिहा** |  |
| **Age in years** |  | **तुमचे वय (वर्षे)** |  |
| **Gender** |  | **लिंग** |  |
| **Kindly choose one answer to the best of your knowledge and put a tick-mark ‘✓’ on it.** | | **खालील प्रश्नाचे तुमच्या मते योग्य ते उत्तर द्या. तुमच्या मते योग्य उत्तरावर ‘✓’ अशी खूण करा.** | |
| **How much has your mother studied?** | A. She cannot read and write (not educated) B. Between 1st and 4th standard C. Between 5th and 9th standard D. 10th standard completed E. 12th standard completed F. Has a Bachelor degree (Such as BA) G. Has a Postgraduate degree (Such as MA) | **आईचे शिक्षण किती झाले आहे?** | A. लिहिता वाचता येत नाही (अशिक्षित) B. इयत्ता १ली ते ४थी च्या दरम्यानचे शिक्षण C. इयत्ता ५वी ते ९वी च्या दरम्यानचे शिक्षण D. १०वी पास E. १२वी पास F. पदवीचे शिक्षण घेतले आहे (जसे की बी.ए.) G. पदव्युत्तर शिक्षण घेतले आहे (जसे की एम.ए.) |
| **What is the occupation of your mother?** | A. Farmer  B. Daily labour  C. Job  D. Housewife E. Runs her own business F. Other:(Please write) _______________ | **आई काय काम करते?** | A. स्वतःच्या शेतीत काम करते  B. रोजची मजुरी करते  C. नोकरी करते D. गृहिणी आहे E. स्वतःचा व्यवसाय आहे F. इतर: (कृपया लिहा) _______________ |
| **How much has your father studied?** | A. He cannot read and write (He is not educated) B. Between 1st and 4th standard C. Between 5th and 9th standard D. 10th standard completed E. 12th standard completed F. Has a Graduate degree (such as BA) G. Has a Postgraduate degree (Such as MA) | **वडिलांचे शिक्षण किती झाले आहे?** | A. लिहिता वाचता येत नाही (अशिक्षित) B. इयत्ता १ली ते ४थी च्या दरम्यानचे शिक्षण C. इयत्ता ५वी ते ९वी च्या दरम्यानचे शिक्षण D. १०वी पास E. १२वी पास F. पदवीचे शिक्षण घेतले आहे (जसे की बी.ए.) G. पदव्युत्तर शिक्षण घेतले आहे (जसे की एम.ए.) |
| **What is the occupation of your father?** | A. Farmer  B. Daily labour  C. Job D. Owns a business E. Other: Please write) _______________ | **वडिल काय काम करतात?** | A. स्वतःच्या शेतीत काम करतात B. रोजची मजुरी करतात  C. नोकरी करतात D. स्वतःचा व्यवसाय आहे E. इतर:(कृपया लिहा) _______________ |
| **What is your religion?** | A. Hindu B. Muslim  C. Christian D. Jain E. Buddhist F. Parsi G. Jew H. Other: (Please write): __________ | **तुमचा धर्म कोणता?** | A. हिंदू B. मुस्लीम  C. ख्रिश्चन D. जैन E. बौद्ध F. पारशी G. ज्यू H. इतर:(कृपया लिहा) _____ |
| **Which caste category do you belong to? If you do not know the category, please write the name of your caste here:________** | A. Open category B. Scheduled castes (SC) C. Scheduled tribes (ST) D. Other backward castes (OBC) E. Other: (Please write):__________ | **तुमची जात कोणती?  ___________ (येथे तुमची जात लिहा लिहा किंवा दिलेल्यापैकी योग्य पर्यायावर ‘✓’ खूण करा)** | A. खुला वर्ग (ओपन/Open) B. अनुसूचित जाती (SC) C. अनुसूचित जमाती (ST) D. अन्य मागासवर्गीय (OBC) E. इतर:(कृपया लिहा) _________ |
| **Kindly choose one answer to the best of your knowledge and put a tick-mark ‘✓’ on it.** | | **खालील प्रश्नाचे तुमच्या मते योग्य उत्तर द्या. योग्य त्या उत्तरावर ‘✓’ अशी खूण करा.** | |
| **Do you have electricity in your house?** | A) Yes B) No | **तुमच्या घरात वीज आहे का?** | A. होय B. नाही |
| **What kind of drinking water facility do you have in your house?** | A. Piped water B. We need to use public water source C. From a well / hand-pump or borewell D. We bring it directly from a river or a water reservoir near our house | **तुमच्या घरात पिण्याच्या पाण्याची कोणती सोय आहे?** | A. घरात पाईपने पाणी येते B. सार्वजनिक नळाचे आणतो C. विहीरीतून / हातपंपाचे आणतो D. घराजवळच्या नदी किंवा तलावातून पाणी आणतो |
| **What kind of toilet facility do you have?** | A. We have a toilet inside our house B. We have a toilet built outside our house C. We use public toilet D. Don’t have a toilet. We do open defecation. | **तुमच्या घरात शौचालयाची/ संडासाची कोणती सोय आहे?** | A. घरात संडास आहे B. घराच्या बाहेर स्वतःचा संडास बांधलेला आहे C. सार्वजनिक संडास वापरतो D. घरात संडास नाही. उघड्यावर जावे लागते |
| **What kind of house do you have?** | A. Kachcha house (Made from grass or hay or mud) B. Semi-pucca (combination of grass/mud.hay and some bricks) C. Pucca (Built of cement/bricks with walls and a roof) | **तुमचे घर कोणत्या प्रकारचे आहे?** | A. कच्चे घर (गवताचे) B. अर्धे-पक्के C. पक्के (सिमेंट व विटांनी बांधलेल्या भिंती व छप्पर असलेले) |
| **How many rooms does your house have? (apart from bathroom and toilet)** | A. One  B. Two C. Three or more | **तुमच्या घरास किती खोल्या आहेत? (न्हाणीघर आणि संडास सोडून)** | A. एक B. दोन C. तीन किंवा तीनपेक्षा जास्त |
| **Do you have an electric fan in your house?** | A) Yes B) No | **तुमच्या घरात विजेवर चालणारा पंखा आहे का?** | A. होय B. नाही |
| **Do you have a radio or transistor or CD player in your house?** | A) Yes B) No | **तुमच्या घरात रेडियो किंवा ट्रान्सीस्टर किंवा सी डी प्लेयर आहे का?** | A. होय B. नाही |
| **Do you have a sewing machine at your home?** | A) Yes B) No | **तुमच्या घरात शिवणाचे मशीन आहे का?** | A. होय B. नाही |
| **Do you have a TV in your house?** | A) Yes B) No | **तुमच्या घरात टीव्ही आहे का?** | A. होय B. नाही |
| **Do you have a landline telephone in your house?** | A) Yes B) No | **तुमच्या घरात टेलीफोनचे कनेक्शन आहे का?** | A. होय B. नाही |
| **Does anyone in your family has a cell-phone (mobile phone)?** | A) Yes B) No | **तुमच्या घरात कुणाकडे मोबाईल फोन आहे का?** | A. होय B. नाही |
| **Do you own a bicycle?** | A) Yes B) No | **तुमच्या घरात सायकल आहे का?** | A. होय B. नाही |
| **Do you own a two-wheeler (bike or moped)?** | A) Yes B) No | **तुमच्या घरात दुचाकी गाडी (मोटरसायकल किंवा स्कूटी) आहे का?** | A. होय B. नाही |
| **Do you own a four-wheeler (jeep or car)?** | A) Yes B) No | **तुमच्या घरात जीप किंवा चारचाकी गाडी (कार) आहे का?** | A. होय B. नाही |
| **Do you own a tractor?** | A) Yes B) No | **तुमच्या घरात ट्रक्टर आहे का?** | A. होय B. नाही |
| **Do you own a computer or a laptop?** | A) Yes B) No | **तुमच्याकडे कम्प्युटर अथवा लॅपटॉप आहे का?** | A. होय B. नाही |
| **Do you own a washing machine?** | A) Yes B) No | **तुमच्याकडे धुणे धुण्याचे यंत्र (वॉशिंग मशीन) आहे का?** | A. होय B. नाही |
| **Which of the following do you use for cooking?** | A. Kerosene B. Gas cylinder or piped gas  C. Wood or chulha D. Other (Please write): __________ | **तुमच्या घरात स्वयंपाकाकरिता काय वापरतात?** | A. केरोसीन B. गॅस सिलेंडर किंवा गॅसचा पाईप C. लाकडाचे सरपण किंवा मातीची चूल D. इतर (कृपया लिहा): ____________ |
| **Kindly choose one answer to the best of your knowledge and put a tick-mark ‘✓’ on it.** | | **कृपया तुमच्या माहितीनुसार योग्य तो पर्याय निवडा व त्यावर ‘✓’ अशी खूण करा.** | |
| **According to you, what does menstruation or MC or periods mean?** | A. Bleeding that occurs among both men and women every month B. Bleeding that occurs every month among women between age-group of 11-45 years C. Don’t know D. Other: (please write):__________________________________ | **तुमच्या मते मासिक पाळी किंवा MC किंवा अंगावरून जाणे म्हणजे काय?** | A. स्त्री व पुरुषांमध्ये दर महिन्याला रक्तस्राव होणे B. स्त्रियांमध्ये वयाच्या साधारण ११ वर्षानंतर ते वयाच्या साधारण ४५ वर्षापर्यंत दर महिन्याला स्त्रियांना रक्त जाते C. माहित नाही D. इतर (कृपया लिहा): ________ |
| **What do you think happens in menstruation or MC or periods?** | A. Blood comes out through vagina among women B. Blood comes out through urinary opening among women C. Blood comes out through urinary opening among women and men D. Don’t know | **तुमच्या मते मासिक पाळीमध्ये खालीलपैकी कोणती गोष्ट होते?** | A. स्त्रियांना गर्भाशयाच्या मुखातून रक्त जाते होतो B. स्त्रियांना शौचाच्या जागेतून रक्त जाते C. पुरुष व स्त्रियांना शौचाच्या जागेतून रक्त जाते D. माहित नाही E. इतर (कृपया लिहा): ________________ |
| **According to you, when does menstruation / MC/ periods usually start in girls?** | A. Since birth B. Approximately at the age of 11 years.  C. When girls get married D. Don’t know | **मासिक पाळी कोणत्या वयात सुरू होते?** | A. जन्मापासून B. साधारण ११ व्या वर्षापासून C. लग्नानंतर D. माहित नाही |
| **Have you ever asked any question regarding menstruation or MC or periods to any of your family members?** | A. Yes B. No | **घरातील कोणत्याही व्यक्तीला मासिक पाळी विषयी तुम्ही कधी प्रश्न विचारला आहे का?** | A. होय B. नाही |
| **(if ever asked), Has any family member avoided answering to your question related to menstruation or MC or periods?** | A. Yes B. No | **मासिक पाळी संदर्भात तुम्ही विचारलेल्या प्रश्नाला घरातील कुणी उत्तर देण्याचे टाळले आहे का?** | A. होय B. नाही |
| **Was there any session in your school where you were given an information on menstruation?** | A. Yes B. No | **शाळेत मासिक पाळीविषयी माहिती देणारा तास झाला का?** | A. होय B. नाही |
| **Do you find comfortable talking about menstruation-related queries to your teacher?** | A. Yes, I can ask my queries to any teacher B. Yes but only to a teacher of same gender  C. No, I feel uncomfortable to ask my queries to any teacher | **मासिक पाळीविषयी काही शंका असल्यास तुम्ही त्या शिक्षकांना विचारता का?** | A. हो, मी मासिक पाळीविषयी शंका कोणत्याही शिक्षकांना विचारू शकते B. हो, परंतु ते शिक्षक की शिक्षिका आहेत त्यावर ठरेल  C. नाही, कुणालाच विचारू शकत नाही |
| **Do you think boys should know about menstruation (periods or MC)?** | A. Yes B. No | **मुलाना मासिक पाळीविषयी माहिती असावी असे तुम्हाला वाटते का?** | A. होय B. नाही |
| **Do you find menstruation a curse for girls and women?** | A. Yes B. No | **मासिक पाळी हा मुलीना आणि स्त्रियांना मिळालेला शाप आहे, असे तुम्हाला कधी वाटते का?** | A. होय B. नाही |
| **Do you think girls/women are dirty when they are having periods?** | A. Yes B. No | **मासिक पाळी सुरू असताना मुली/स्त्रिया अस्वच्छ/घाण असतात असे तुम्हाला वाटते का?** | A. होय B. नाही |
| **Are women impure during periods?** | A. Yes, they are acceptable because women are impure during periods B. No, they are not acceptable because women are not impure during periods | **मासिक पाळीत स्त्री ला स्पर्श न करणे तुमच्या मते योग्य की अयोग्य?** | A. बरोबर आहे कारण पाळी सुरु असताना बायका अपवित्र असतात B. बरोबर नाही कारण पाळी सुरु असताना बायका अपवित्र नसतात |
| **Has your menstruation or periods or MC started?** | A. Yes B. No | **तुम्हाला पाळी सुरू झाली आहे का?** | A. होय B. नाही |
| **Did anyone talk to you about using a cloth or a pad when you talked to someone for the first time about your menses?** | A. Yes, I was told how to use cloth / pad  B. Nothing was told to me | **सगळ्यात पहिल्यांदा पाळी आल्याचे सांगितल्यावर तुमच्याशी कुणी कापड अथवा पॅड वापरण्याविषयी बोलले का?** | A. हो, कापड वा पॅड कसे वापरायचे हे मला सांगितले होते  B. नाही, कापड अथवा पॅड वापरण्याविषयी माझ्याशी कुणी बोलले नाही |
| **Were you told not to go to shrine or praying room or touch god’s idol or do any religious activity during periods?** | A. Yes B. No | **पाळी सुरू असताना देवघरात जायचे नाही, धार्मिक स्थळांना भेट द्यायची नाही असे तुम्हाला सांगितले का?** | A. होय B. नाही |
| **Do men in the house usually know when you are menstruating (having periods)?** | A. Yes B. No | **तुम्हाला पाळी चालू असते तेव्हा ते घरातील पुरुषांना माहित असते का?** | A. होय B. नाही |
| **Were you told by someone to hide the cloth or pad from men that you use during your periods?** | A. Yes B. No | **घरातील पुरुषांना पाळीत वापरण्याचे कापड अथवा पॅड दिसता कामा नये, असे तुम्हाला सांगितले होते का?** | A. होय B. नाही |
| **Were you told to sit at a distance from others in the house while having lunch or dinner during menstruation?** | A. Yes B. No | **पाळी सुरू असताना जेवताना इतरांपासून लांब बसायचे असे तुम्हाला सांगितले आहे का?** | A. होय B. नाही |
| **Were you told not to touch pickle, paapad or plants during periods?** | A. Yes B. No | **पाळी सुरू असताना पापड, लोणची किंवा झाडांना हात लावायचा नाही, असे तुम्हाला सांगितले का?** | A. होय B. नाही |
| **Were you told not to speak to boys or men after your periods started?** | A. Yes B. No | **पाळी सुरू झाल्यावर मुलांशी अथवा पुरुषांशी बोलायचे नाही, असे तुम्हाला सांगितले का?** | A. होय B. नाही |
| **Were you told that girls or women should not be touched when they are having periods?** | A. Yes B. No | **पाळी सुरू असताना बाईला हात लावायचा नाही, असे तुम्हाला सांगितले आहे का?** | A. होय B. नाही |
| **Have you ever experienced extreme abdominal pain during menses?** | A. Yes B. No | **पाळीच्या वेळेस कधी पोटात कधी खूप दुखले का?** | A. होय B. नाही |
| **Have you ever experienced itching near private parts?** | A. Yes B. No | **कधी जांघेच्या भागात खाज येण्याचा त्रास झाला का?** | A. होय B. नाही |
| **Have you ever experienced a foul smell near your private parts?** | A. Yes B. No | **तुमच्या जांघेच्या भागातून कधी उग्र किंवा घाण वासाचा त्रास तुम्हाला झाला का?** | A. होय B. नाही |
| **Have you ever experienced white discharge* from private parts? (*White color liquid that comes out in excess from the birth canal)** | A. Yes B. No | **अंगावरून कधी पांढरे पाणी/ स्राव गेले का?** | A. होय B. नाही |
| **Did you ever get periods more than once in about a month?** | A. Yes B. No | **एका महिन्यात एका पेक्षा जास्त वेळा पाळी आली, असे कधी घडले का?** | A. होय B. नाही |
| **Did you ever miss your periods?** | A. Yes B. No | **कधी तुमची पाळी चुकली का?** | A. होय B. नाही |
| **Did you ever feel giddy or weak during your periods?** | A. Yes B. No | **पाळीच्या वेळी खूप अशक्तपणा आला किंवा चक्कर आली असे झाले का?** | A. होय B. नाही |
| **Did you ever miss your school because of the periods?** | A. Yes B. No | **पाळी सुरु असल्यामुळे कधी तुमची शाळा चुकली का?** | A. होय B. नाही |
| **Do you feel stressed during your periods about the possibility of getting stains on your clothes?** | A. Yes B. No | **पाळीच्या दिवसात डाग पडेल याचा तुमच्या मनावर ताण असतो का?** | A. होय B. नाही |
